# Supplementary material for: Identification of leukemic and pre-leukemic stem cells by clonal tracking from single-cell transcriptomics
Source: Nat Commun. 2021 Mar 1;12:1366. doi: 10.1038/s41467-021-21650-1 (PMC7921413; doi:10.1038/s41467-021-21650-1)
Supplement: Supplementary file 1 — Supplementary Information [file 41467_2021_21650_MOESM1_ESM.pdf]

Supplementary information to *Identification of leukemic and pre-leukemic stem cells by clonal tracking from single-cell transcriptomics*

Lars Velten, Benjamin A. Story, Pablo Hernández-Malmierca, et al.

Contains Supplementary Figures 1-10, Supplementary Table 1 and Supplementary References

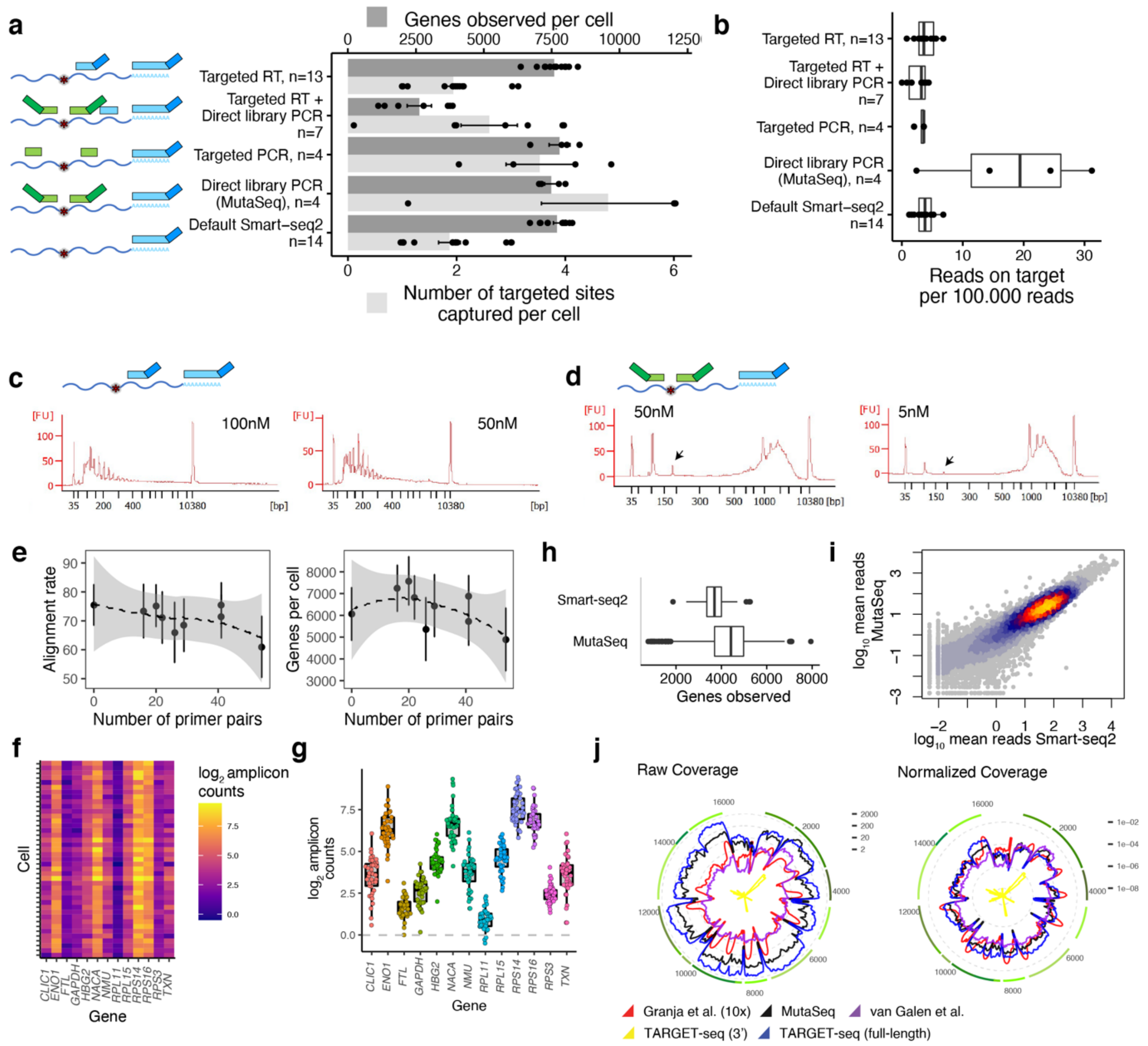

Supplementary Figure 1. Development of MutaSeq. See also Figure 1.

a. Comparison of different strategies for targeting 14 genomic sites of interest during Smart-seq2 library preparation; see Supplementary Data 4 for primers used. In the targeted RT protocol, a reverse transcription primer carrying the ISPCR sequence was placed downstream of the sites of interest. In the targeted RT+ direct library PCR protocol, a targeted RT primer without ISPCR sequence was used in conjunction with targeting primers included during PCR. In the targeted PCR protocol, PCR primers were used to generate amplicons of 250-350 bases, whereas in the direct library PCR protocol, shorter amplicons were used and primers were fused to Nextera sequencing adapters; see also Figure 1b. K562 cells were sequenced with each protocol and the number of genes observed per cell as well as the number of target sites

covered was quantified. The number of cells sequenced per condition (n) is indicated in the axis labels. Error bars indicate the standard error of the mean. For all analysis in this panel, reads were down-sampled to 250,000 reads per cell prior to alignment to account for differences in coverage.

b. Boxplot comparing the mean number of reads per target in the different protocols. See Methods section on *Data Visualization* for a definition of boxplot elements. The number of cells sequenced per condition is indicated in the axis labels.

c. Bioanalyzer traces for a protocol targeting sites of interest during RT only. Concentrations correspond to the final concentration of targeting primers in the RT reaction.

d. Bioanalyzer traces for a protocol targeting sites of interest during library amplification PCR (MutaSeq). Arrows highlight the MutaSeq amplicons. Concentrations correspond to the final concentration of targeting primers in the PCR reaction.

e. Primer sets developed for the MutaSeq patients (Supplementary Data 4) were combined in all possible combinations (i.e. P1+P2 primers, P1+P3+P4 primers, etc.) and libraries were generated from n=8 K562 cells each to evaluate the effect of multiplexing primers on alignment rate (left) and number of genes observed (right). Error bars indicate standard deviation.

f. Primer pairs were designed surrounding randomly selected sites on 13 highly-expressed genes in K562 cells (Supplementary Data 4). The MutaSeq protocol was then performed using these primers on n=48 K562 cells. For each gene, the number of reads from MutaSeq amplicons (i.e. complete matches) is shown, after subtracting the average coverage of the surrounding areas outside of the targeted site (i.e. potential background signal). Seven cells with poor alignment rates (below 50%) were removed. n=41 cells are shown.

g. Amplicon counts for 13 genes across 41 cells is shown as boxplots. The points in the overlaid beeswarm plot represent n=41 cells. Same underlying data as used in Supplementary Figure 1f. See Methods section on *Data Visualization* for a definition of boxplot elements.

h. Number of genes observed per cell, across n=206 (Smart-seq2) or n=658 CD34+ (MutaSeq) cells. See Methods section on *Data Visualization* for a definition of boxplot elements.

i. Mean gene expression levels measured by Smart-seq2 are compared to mean gene expression levels measured by MutaSeq. Color reflects point density.

j. Logarithmic coverage of the mitochondrial genome compared between different methods[1–3]. For the plot on the right, coverage was normalized to the number of reads aligning to the transcriptome.

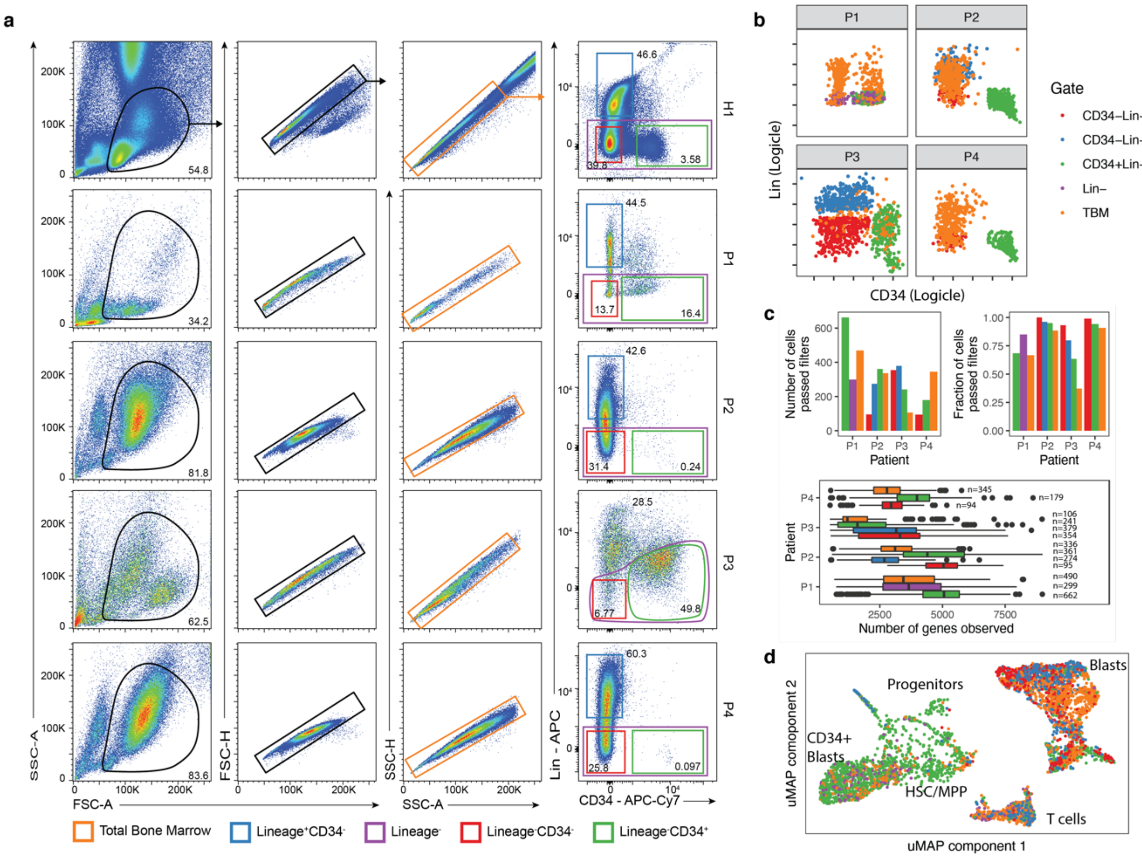

Supplementary Figure 2. FACS sorting schemes and quality control of single-cell RNA-seq data. See also Figure 2+3.

a. Gating schemes used for the different patients.

b. Index values of cells included into the final data set

c. Top left: number of cells from the various gates included into the final data set; see panel a/b for a color scheme. Top right: fraction of cells passing filters, stratified by patient and gate. Bottom: box plots depicting the number of genes observed in cells from the final data set. The number of single cells underlying each box-and-whisker plot (n) is specified in the figure. See Methods section on *Data Visualization* for a definition of boxplot elements.

d. uMAP plot of cells from all individuals, with cells color coded according to their sorting gate. See panel a/b for a color scheme, and main Figures 3a-e and Supplementary Fig. 7a for a more detailed description of the uMAP.

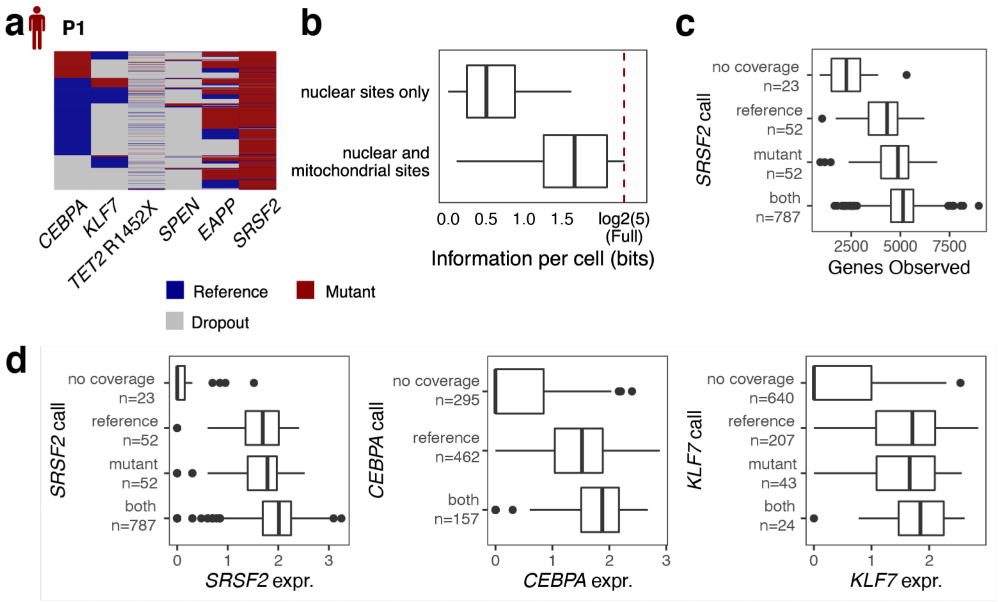

Supplementary Figure 3. Evaluation of nuclear markers for clonal tracking in single-cell RNA-seq data. See also Figure 2.

a. Heatmap depicting mutation calls of nuclear genomic mutations only for P1. Clustering was performed as described in the methods, section *Analysis of mitochondrial mutations and reconstruction of clonal hierarchies*, except that here, only nuclear mutations were included. Rows represent cells.

b. The full specification of clonal identities in case of P1 requires log2(5) bits of information, since there are five main clones (Figure 2e). For each of n=1430 cells, the information available from nuclear genomic sites only, or both nuclear and mitochondrial sites, was quantified as described in the Methods, section *Analysis of mitochondrial mutations and reconstruction of clonal hierarchies.* See Methods section on *Data Visualization* for a definition of boxplot elements.

c. Box plots evaluating the extent to which differences in library quality affect measurements of the mutational status of target genes; data for 914 cells with the HSC/MPP-like, CD34+ AP1-high and CD34+ AP1-low identies from P1 is shown. The number of single cells underlying each box-and-whisker plot (n) is specified in the axis labels. See Methods section on *Data Visualization* for a definition of boxplot elements.

d. Like panel c, but investigating the effect of the expression of a targeted gene on the observed mutational status. The number of single cells underlying each box-and-whisker plot (n) is specified in the axis labels. See Methods section on *Data Visualization* for a definition of boxplot elements.

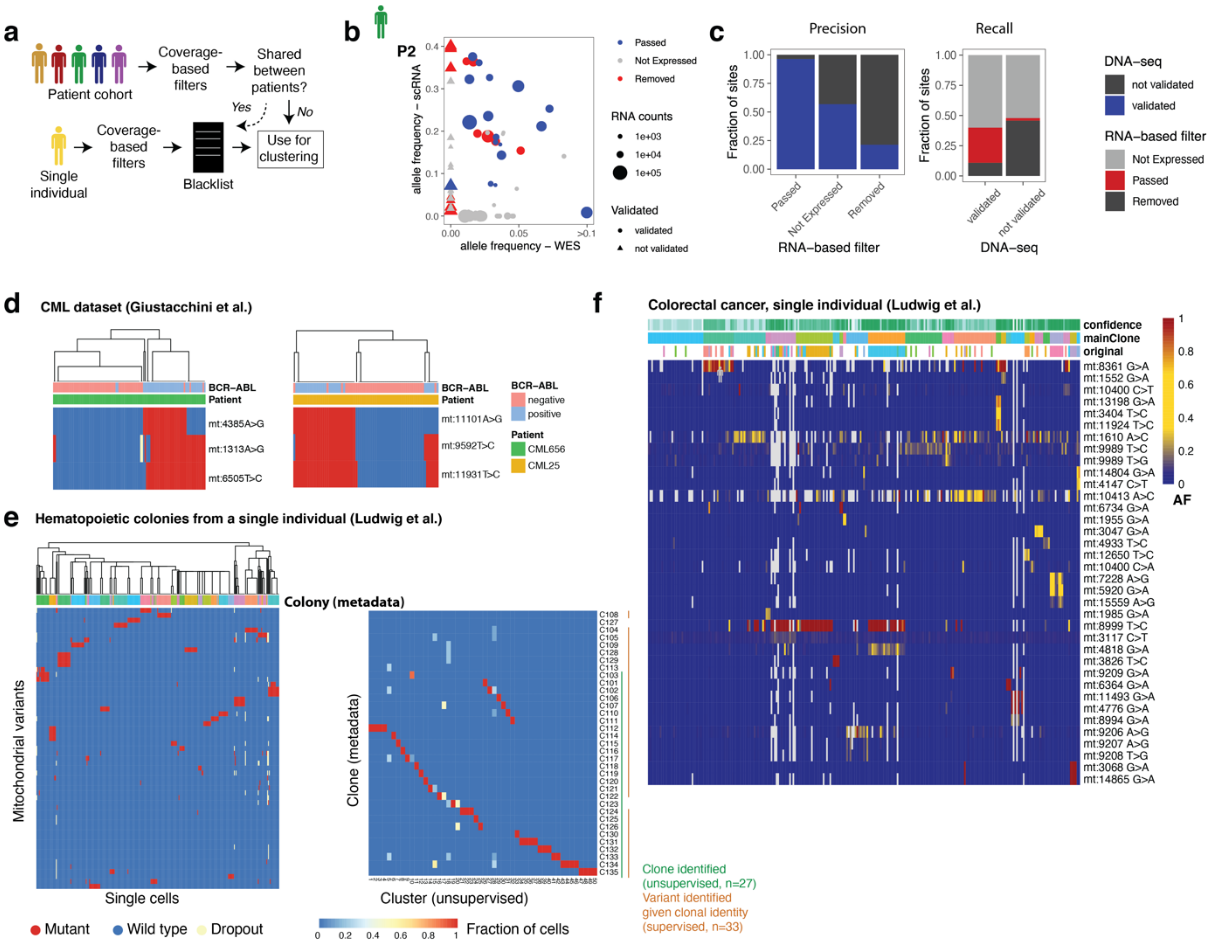

Supplementary Figure 4. Calling of mitochondrial somatic variants in the absence of a DNA-based reference. See also Figure 2. For an implementation and for reproducing the computations, see the package vignettes of the mitoClone package.

a. Overview of the computational strategy used. In the case of data from a group of individuals, sites were filtered based on coverage and annotated as 'mutant' if a specified fraction of reads deviates from the reference allele. Sites were then excluded as likely RNA editing events if the same mutation was observed in more than one individual. Alternatively, in the case of data from a single individual, similar coverage based filtered were applied and data was then filtered against a blacklist created from a cohort.

b. Allele frequencies and coverage of mitochondrial mutations from P2 in single-cell RNA-seq data compared to whole exome sequencing data (WES). Sites with less than 10 reads per cell in RNA-seq were classified as not expressed. Variants that were observed in WES were classified as validated (circles), other variants were classified as not validated (triangles). The low correlation between the two datasets is likely due to different starting cell populations (WES: Total bone marrow, single cell RNA-seq: enriched for CD34+ cells), and data are only used for qualitative statements (presence/absence of mutations).

c. Bar charts summarizing the classifications from panel b. Left, mutation sites are split by their label based on the mitoClone pipeline; right, sites are split by whether they were detected in WES.

d. De novo variant calling and clustering of a CML patient dataset. Data from ref. [4] were processed and clustered with the mitoClone package. The same variant filtering approach used on the patients from our study was used. Thereby, two patients with substantial mitochondrial variability were identified and in both cases clones associated with the BCR-ABL mutation were resolved in an unsupervised manner. The analysis by ref. [5] had missed one of these patients, did not achieve an unsupervised separation of BCR-ABL+ and BCR-ABL- cells in either case (Figure 7G in ref. [5]), and instead relied on stratifying cells by the existing BCR-ABL label (Figure 7J in ref. [5]).

e. De novo variant calling and clustering of single cells from hematopoietic colonies derived from a single individual. Data from Figure 5 of ref. [5] were processed and clustered with the mitoClone package. Left panel shows unsupervised clustering of mutations identified by the mitoClone package, right panel quantitatively compares unsupervised clustering and colony labels. 27 of the colonies were identified in an unsupervised manner. The analysis by ref. [5] had identified approximately half that number by unsupervised analyses (their Figure 5E), and using supervised methods identified mitochondrial mutations associated with 33 clones (their Figure 5H).

f. De novo variant calling and clustering of single cells from a single colorectal cancer patient. Data from Figure 7 of ref. [5] were processed and clustered with the mitoClone package. Clustering structure obtained by PhISCS is shown and compared to the clustering presented in ref. [5] (row labeled 'original'), which was based on variant filtering using a DNA-seq based reference. Despite the different filtering approaches, our unsupervised clustering separated the clusters identified by Ludwig et al. and identified additional variability.

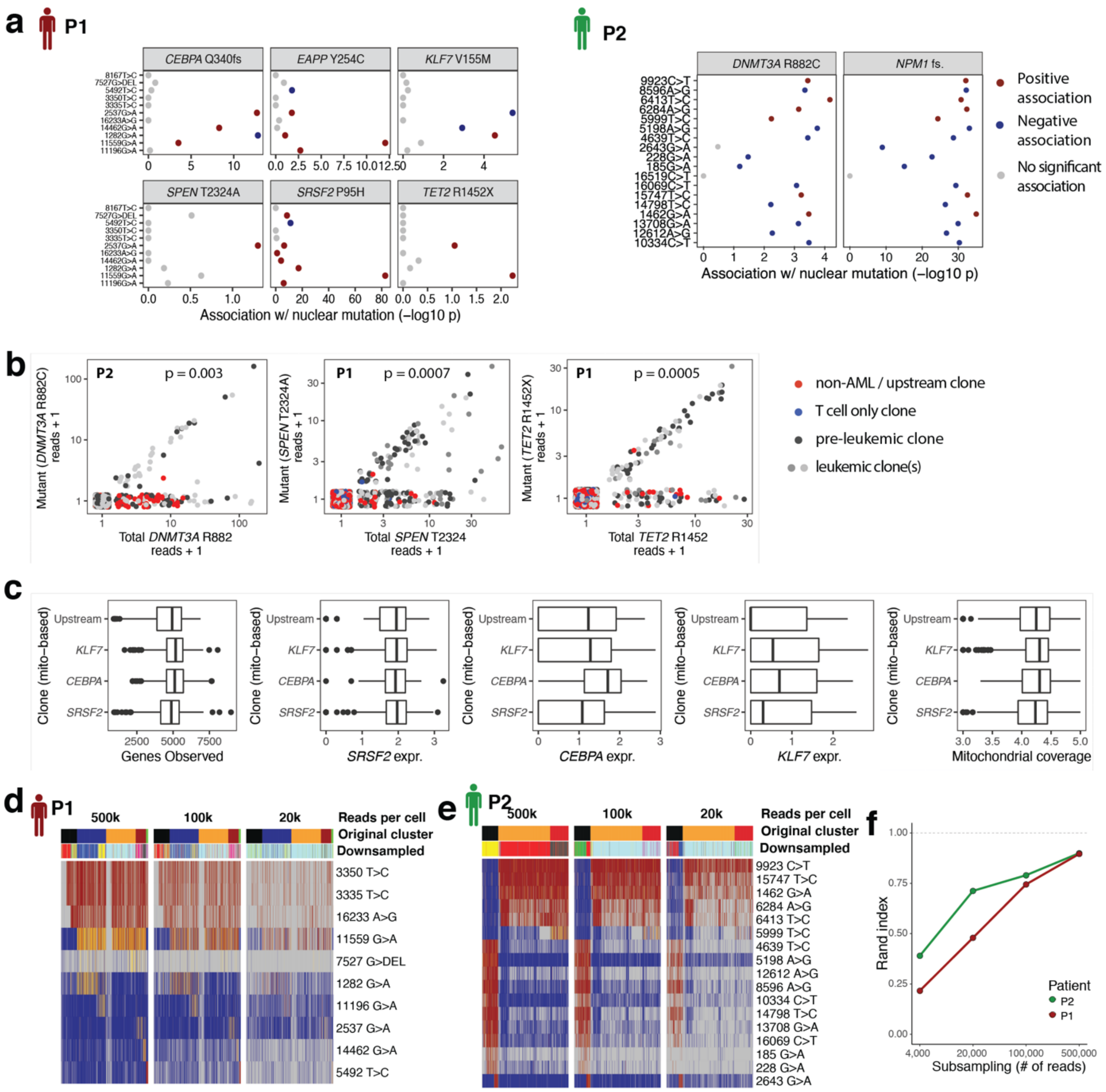

Supplementary Figure 5. Evaluation of mitochondrial markers for clonal tracking in single-cell RNA-seq data. See also Figure 2.

a. Association between various mutations (y axis) and nuclear mutations (panels) across n=1430 cells from P1 or n=1066 cells from P2. P-values are from a two-sided Fisher test.

b. Association between the lowly covered mutations in *DNMT3A*, *SPEN* and *TET2* with clonal identity. Scatter plot depicts total coverage on the site of interest (x axis) and the number of mutant reads (y axis) across n=1066 cells from P2 (left panel) or n=1430 cells from P1 (central and right panel). Note that jitter was added in the x and y direction to avoid overplotting. P-values are from a chi-square test comparing a model where the probability of detecting at least one mutant read was modelled as a function of total coverage (null model), or a function of total coverage and identity as a non-AML/upstream clone (alternative model).

c. Box plots evaluating the extent to which differences in marker gene expression and library quality affect clonal assignments when using mitochondrial marker mutations; data for 914 cells with the HSC/MPP-like, CD34+ AP1-high and CD34+ AP1-low identies from P1 is shown.

Clone labeled SRSF2 refers to the pre-leukemic clone from main figure 2 while clones labeled KLF7 and CEBPA refer to the leukemic clones. Number of single cells: n=262 (SRSF2 clone), n=155 (CEBPA clone), n=378 (KLF7 clone) and n=113 (Upstream clone). See Methods section on *Data Visualization* for a definition of boxplot elements.

d. Effect of read depth on mitochondrial clusters. Clusters obtained from mitochondrial sites only were computed at full read depth (row "Original clusters") and are compared to clusters obtained using the same methodology from data were single cells were down-sampled to read depths of 500k, 100k, or 20k per cell ("Downsampled"). Data from Patient 1 is shown.

e. Like panel c, but for patient 2 (P2).

f. Original clustering result and down-sampled clustering result are compared quantitatively using the Rand index.

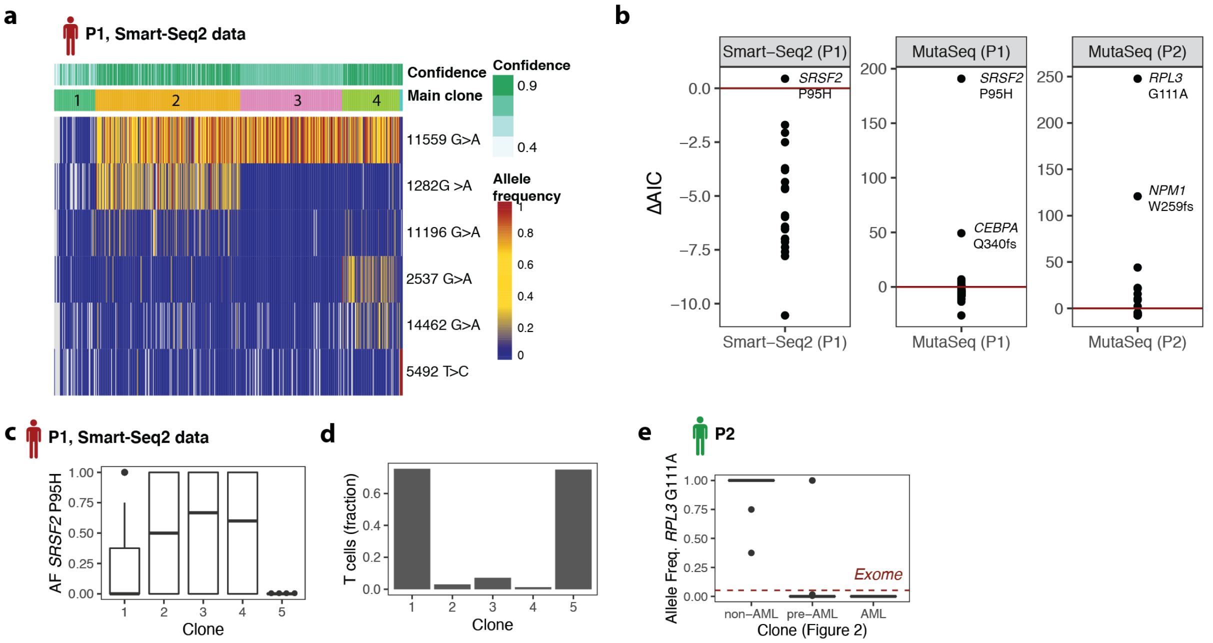

Supplementary Figure 6. De novo calling and characterization of clones. See also Figure 2. For an implementation and for reproducing the computations, see the package vignettes of the mitoClone package.

a. Unsupervised clustering of mitochondrial mutations identified from a Smart-seq2 dataset of n=672 cells from patient P1.

b. De novo identification of nuclear somatic variants associated with the clonal labels from panel a. Difference in Aikake's Information Criterion (AIC) is shown for a comparison between a model where allele frequencies are the same across all cells, and a model where allele frequencies differ between clones. Red line highlights the intercept. See Methods section *Analysis of mitochondrial mutations and reconstruction of clonal hierarchies*.

c. Boxplot of single-cell allele frequencies for the *SRSF2* P95H mutation summarized between clones (Smart-seq2 data). Number of single cells: n=57 (clone 1), n=201 (clone 2), n=141 (clone 3), n=80 (clone 4), n=4 (clone 5). See Methods section on *Data Visualization* for a definition of boxplot elements.

d. Bar plot of contribution of clones to T cells identified from unsupervised clustering of gene expression data (see also Figure 3).

e. Boxplot of single-cell allele frequencies for the *RPL3* mutation (COSV53365368) summarized between clones (MutaSeq data). Red dashed line highlights the allele frequency of the mutation identified in exome sequencing. Number of single cells: n=200 (non-AML), n=641 (pre-AML), n=225 (AML).

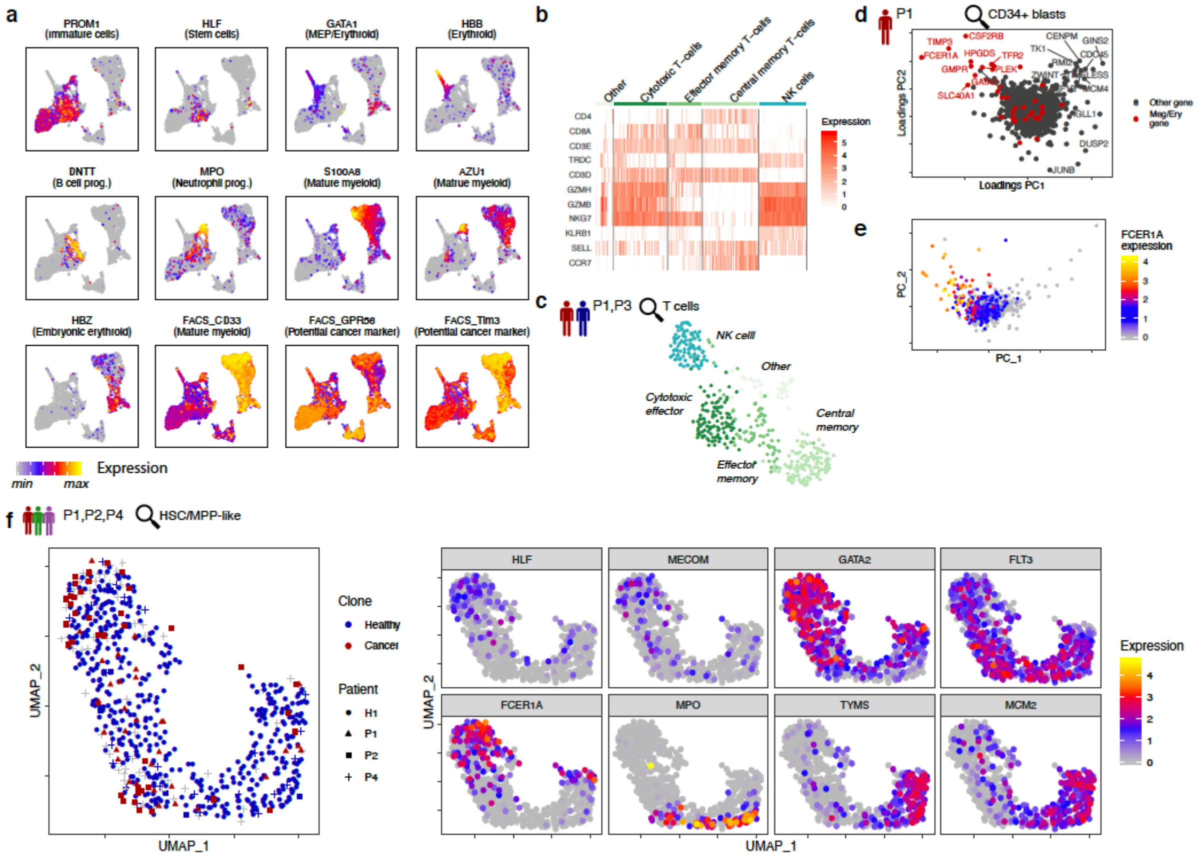

Supplementary Figure 7. Analysis of single-cell gene expression data. See also Figure 3.

a. Expression of marker genes on the uMAP from Figure 3. Top rows: log-normalized mRNA expression. Bottom row (labels preceded FACS_): Logicle transformed FACS index values.

b. Heatmap depicting the expression of selected marker genes across T-cells from patients P1 and P3. Columns are cells.

c. uMAP representation of all T-cells from patients P1 and P3. Data from these cells only were integrated using MNN[6] and visualized in two dimensions using uMAP. Patients P2 and P4 were omitted from this analysis due to an insufficient number of T cells. Colors denote cluster identity.

d. Loadings plot of a principal component analysis of all CD34+ blasts from Patient P1. Genes associated with erythroid or megakaryocytic priming[7] are highlighted in red.

e. Scores plot of a principal component analysis of all CD34+ blasts from Patient P1. Expression levels of the FCER1A gene are color-coded.

f. uMAP representation of all cells with a healthy HSPC-like gene expression signature from patients P1, P2 and P4. These include both healthy and pre-(leukaemic) clones, see figure 4.

Data from these cells only were integrated using MNN[6] and visualized in two dimensions using uMAP. Left panel: clonal identity is highlighted, using the same strategy as in Figure 4e. Right panels: Point color represents the expression of genes involved in differentiation (*MPO* for myeloid differentiation, *FCER1A* for erythroid/megakaryocytic differentiation) and cell cycle (*TYMS, MCM2*).

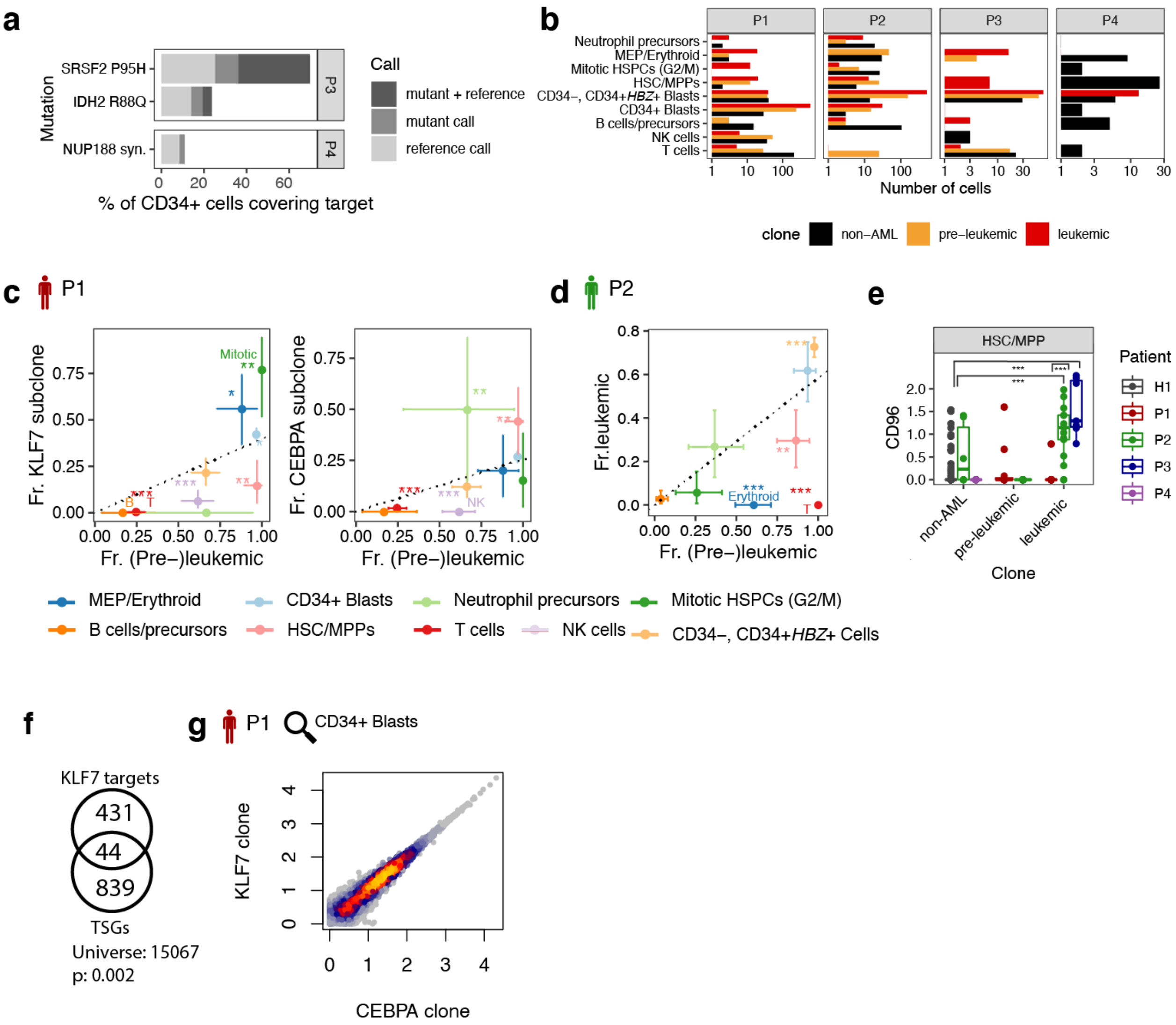

Supplementary Figure 8. Analysis of single-cell clonal tracking data. See also Figures 4 and 5.

a. Bar chart depicting the percentages of cells with coverage of the mutations used for annotating clones in P3 and P4.
b. Bar chart depicting the absolute cell numbers of the different clones in the different cell populations. Cells were assigned to clones as in Figure 4e.
c. Scatter plot depicting the fraction (Fr.) of (pre-)leukemic cells in relation to the fraction of cells from the sub-clones for P1. Dotted line indicates the mean ratio across all cells, error bars denote 95% confidence intervals from a beta distribution, and asterisk indicate significant deviation from the mean ratio, as follows: *: $p < 0.05$; **: $p < 0.01$; ***: $p < 0.001$. p-values are from a two-sided binomial test and were not adjusted for multiple testing. Only cells with a confident assignment to clones (likelihood > 0.8, see Methods section *Analysis of mitochondrial*

mutations and reconstruction of clonal hierarchies) are included. See figure source data for number of single cells underlying each group.

d. As Supplementary Figure 8c but investigating instead the second patient (P2). See figure source data for number of single cells underlying each group.

e. Boxplots comparing the normalized, scaled expression levels of *CD96* between cells with evidence of originating from the non-leukemic clone(s), and cells with evidence of originating from the leukemic or pre-leukemic clones. Cells were assigned to clones as in Figure 4e. Asterisk denote p-values from a two-sided Wilcoxon test for relevant comparisons: leukemic P2 vs. all non-leukemic: 1.7e-40; leukemic P3 vs. all non-leukemic: 4.1e-31; leukemic P1 vs. all other leukemic: 6.4e-8. See figure source data for number of single cells underlying each group. See Methods section on *Data Visualization* for a definition of boxplot elements.

f. Venn diagram depicting the number of open chromatin regions containing a KLF7 binding site, the number of open chromatin regions proximal to tumor suppressor genes from[8], and their overlap. N=15067 open chromatin regions were identified from single-cell ATAC-seq data of human CD34+ bone marrow cells[9]. P value is from a hypergeometric test.

g. Scatter plot comparing the log10 mean gene expression levels in CD34+ blasts from the *CEBPA*-mutated clone, and CD34+ blasts from the KLF7 mutated clone. N=15,451 genes with a mean expression of at least 1 in either population are shown. Color represents point density.

	with mitoClone package					no mitoClone package	
	MutaSeq	SmartSeq	TARGET-seq Full l.	TARGET-seq 3'	GoT-seq	SmartSeq	ATAC-seq
Identification of clones (de novo)	✓✓*	✓✓*	0*	×	×	×	✓✓*
Association of nuclear and mitochondrial variants	✓✓*	0*	0*	×	×	×	×
Identification of clones (known variants)	✓	0	✓✓	✓✓	✓	0	×
Differential expression testing between clones	✓✓*	✓✓*	✓✓	✓✓	×	×	×
Throughput	✓	✓	×	✓	✓✓	✓	✓✓

* If natural mitochondrial somatic variability is present.

Supplementary Figure 9. Overview of capabilities of MutaSeq compared to related methods. Full-l.: full-length. SmartSeq2: ref. [5,10]. TARGET-seq: ref. [2]. GoT-seq: ref. [11]. 0 means theoretically possible, but unproven and/or very limited.

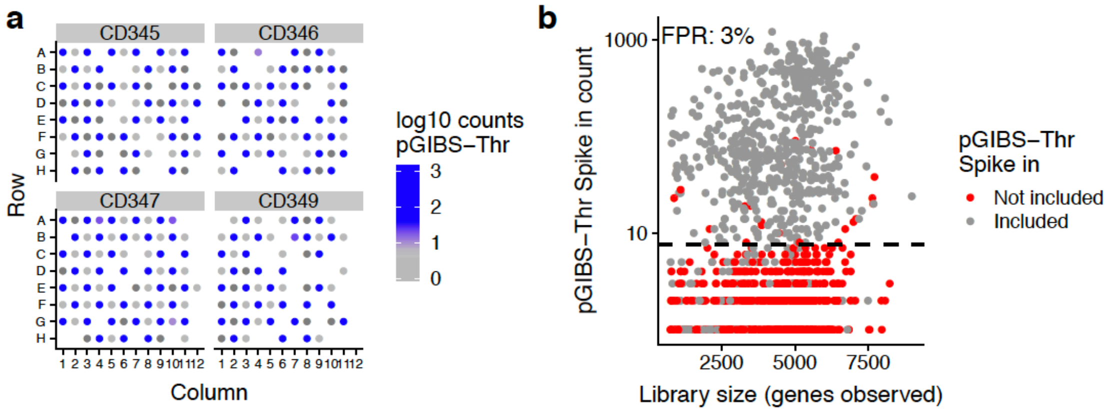

Supplementary Figure 10. Estimation of MutaSeq's false positive rate.

a. To estimate MutaSeq's false positive rate, a polyadenylated in vitro transcript, pGIBS-Thr[12], was added to every second well (A1, A3, B2, B4, etc.) during the P1 experiment. Abundance of the pGIBS-Thr spike-in across wells from four representative plates is shown.

b. Estimation of the false positive rate using the pGIBS-Thr spike-in. Dashed bold line indicates the threshold used for classifying a site as dropout.

Supplementary Table 1. Antibodies used for flow cytometry.

Antigen	Used for	Clone	Fluorochrome	Company	Catalogue No.	Dilution
CD135	Index Sort	4G8	PE	BD Pharmingen	558996	1:20
CD15	Colony Classification	W6D3	Alexa700	BioLegend	323026	1:100
CD19	Colony Classification	HIB19	eFluor 450	eBioscience	48-0199-42	1:80
CD19	Index Sort	HIB19	APC	eBioscience	17-0199-42	1:20
CD20	Index Sort	2H7	APC	BD Pharmingen	559776	1:20
CD235a	Colony Classification/Index Sort	HIR2	APC	BD Pharmingen	551336	1:30
CD33	Colony Classification	WM-53	PE-Cy7	eBioscience	12-0338-42	1:200
CD33	Index Sort	WM53	BV421	BioLegend	303416	1:100
CD34	Colony Classification/Index Sort	4H11	APC-eFluor 780	eBioscience	47-0349-42	1:30
CD38	Index Sort	HIT2	Alexa 700	eBioscience	56-0389-42	1:30
CD4	Index Sort	RPA-T4	APC	BD Pharmingen	555349	1:20
CD41a	Colony Classification	HIP8	FITC	eBioscience	11-0419-42	1:200
CD41a	Index Sort	HIP8	APC	eBioscience	17-0419-42	1:30
CD45	Colony Classification/Mesenchymal sort	HI30	PE	eBioscience	12-0459-42	1:200
CD45RA	Index Sort	HI100	FITC	BioLegend	983002	1:20
CD66b	Colony Classification	G10F5	PerCP/Cy5.5	BioLegend	305108	1:100
CD8	Index Sort	RPA-T8	APC	BD Pharmingen	555369	1:20
CD90	Index Sort	5E10	PE-Cy5	BD Pharmingen	555597	1:20
GPR56	Index Sort	CG4	PE-Cy7	BioLegend	358206	1:20
Tim3	Index Sort	F38-2E2	BV605	BioLegend	345018	1:50
CD105	Mesenchymal sort	43A3	FITC	BioLegend	323204	1:30

Supplementary References

1. Granja, J. M. *et al.* Single-cell multiomic analysis identifies regulatory programs in mixed-phenotype acute leukemia. *Nat. Biotechnol.* **37**, 1458–1465 (2019).
2. Rodriguez-Meira, A. *et al.* Unravelling Intratumoral Heterogeneity through High-Sensitivity Single-Cell Mutational Analysis and Parallel RNA Sequencing. *Mol. Cell* **73**, 1292-1305.e8 (2019).
3. van Galen, P. *et al.* Single-Cell RNA-Seq Reveals AML Hierarchies Relevant to Disease Progression and Immunity. *Cell* **0**, 1–17 (2019).
4. Giustacchini, A. *et al.* Single-cell transcriptomics uncovers distinct molecular signatures of stem cells in chronic myeloid leukemia. *Nat. Med.* **23**, 692–702 (2017).
5. Ludwig, L. S. *et al.* Lineage Tracing in Humans Enabled by Mitochondrial Mutations and Single-Cell Genomics. *Cell* **176**, 1325-1339.e22 (2019).
6. Haghverdi, L., Lun, A. T. L., Morgan, M. D. & Marioni, J. C. Batch effects in single-cell RNA-sequencing data are corrected by matching mutual nearest neighbors. *Nat. Biotechnol.* **36**, 421–427 (2018).

7. Velten, L. *et al.* Human haematopoietic stem cell lineage commitment is a continuous process. *Nat. Cell Biol.* **19**, 271–281 (2017).
8. Zhao, M., Kim, P., Mitra, R., Zhao, J. & Zhao, Z. TSGene 2.0: an updated literature-based knowledgebase for tumor suppressor genes. *Nucleic Acids Res.* **44**, D1023–D1031 (2016).
9. Satpathy, A. T. *et al.* Massively parallel single-cell chromatin landscapes of human immune cell development and intratumoral T cell exhaustion. *Nat. Biotechnol.* **37**, 925–936 (2019).
10. Picelli, S. *et al.* Smart-seq2 for sensitive full-length transcriptome profiling in single cells. *Nat. Methods* **10**, 1096–1098 (2013).
11. Nam, A. S. *et al.* Somatic mutations and cell identity linked by Genotyping of Transcriptomes. *Nature* **571**, 355–360 (2019).
12. Pelechano, V., Wilkening, S., Järvelin, A. I., Tekkedil, M. M. & Steinmetz, L. M. Genome-wide polyadenylation site mapping. *Methods Enzymol.* **513**, 271–96 (2012).
