# Supplementary material for: Identification of leukemic and pre-leukemic stem cells by clonal tracking from single-cell transcriptomics
Source: Nat Commun. 2021 Mar 1;12:1366. doi: 10.1038/s41467-021-21650-1 (PMC7921413; doi:10.1038/s41467-021-21650-1)
Supplement: Supplementary file 3 — Description of Additional Supplementary Files [file 41467_2021_21650_MOESM3_ESM.pdf]

Description of Additional Supplementary Files

Supplementary Data 1. Exome sequencing results. Columns contain genomic position of mutations, reference and alternate alleles, consequence for protein sequence, gene biotype, and allele frequencies in their respective patients' cancer (af_tumor) and healthy (af_normal) exomes. Patients are separated by tab.

Supplementary Data 2. Genes with significant (false discovery rate <0.001) overexpression in the various cell populations. For DE testing, MAST with library quality and patient as covariates was used.

Supplementary Data 3. Results from differential expression test underlying figures 3g, 5b, 5e.

Supplementary Data 4. Primers used for targeting genes of interest with MutaSeq.

Supplementary Data 5. Maximum likelihood trees obtained from different parameter setting of the PhISCS model. See also methods, section Construction of clonal hierarchies. PhISCS model parameters (gene-specific allelic dropout rates) were sampled using a latin hypercube and the maximum likelihood tree was computed. Zip file contains tree structures from 80 sampling runs as png files, as well as a pdf file displaying the parameter distributions.
