# Supplementary figures and images for: Identification of leukemic and pre-leukemic stem cells by clonal tracking from single-cell transcriptomics
Source: Nat Commun. 2021 Mar 1;12:1366. doi: 10.1038/s41467-021-21650-1 (PMC7921413; doi:10.1038/s41467-021-21650-1)

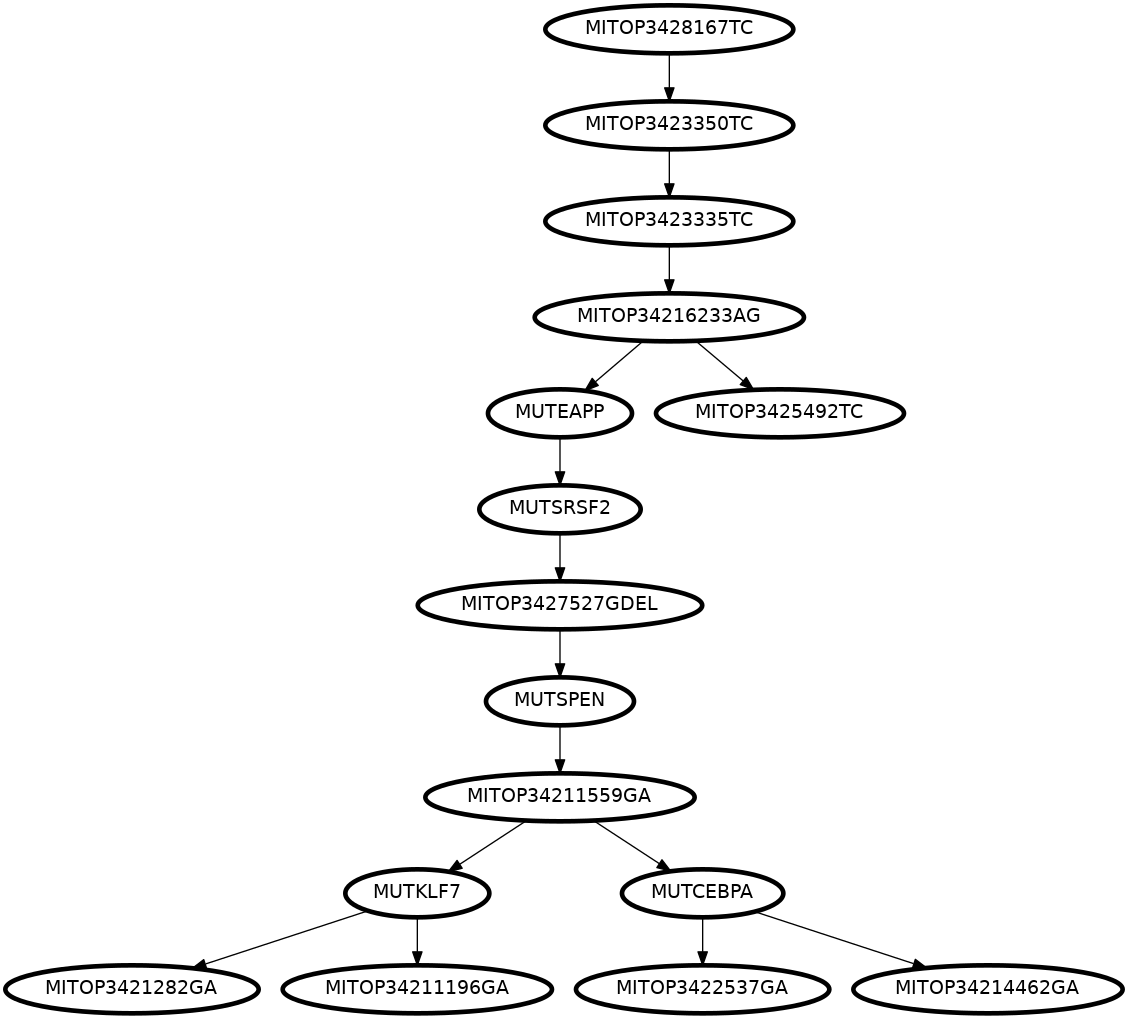

Supplement: Supplementary file 8 — Supplementary Data 5 [file 41467_2021_21650_MOESM8_ESM.zip › P342_001.png]

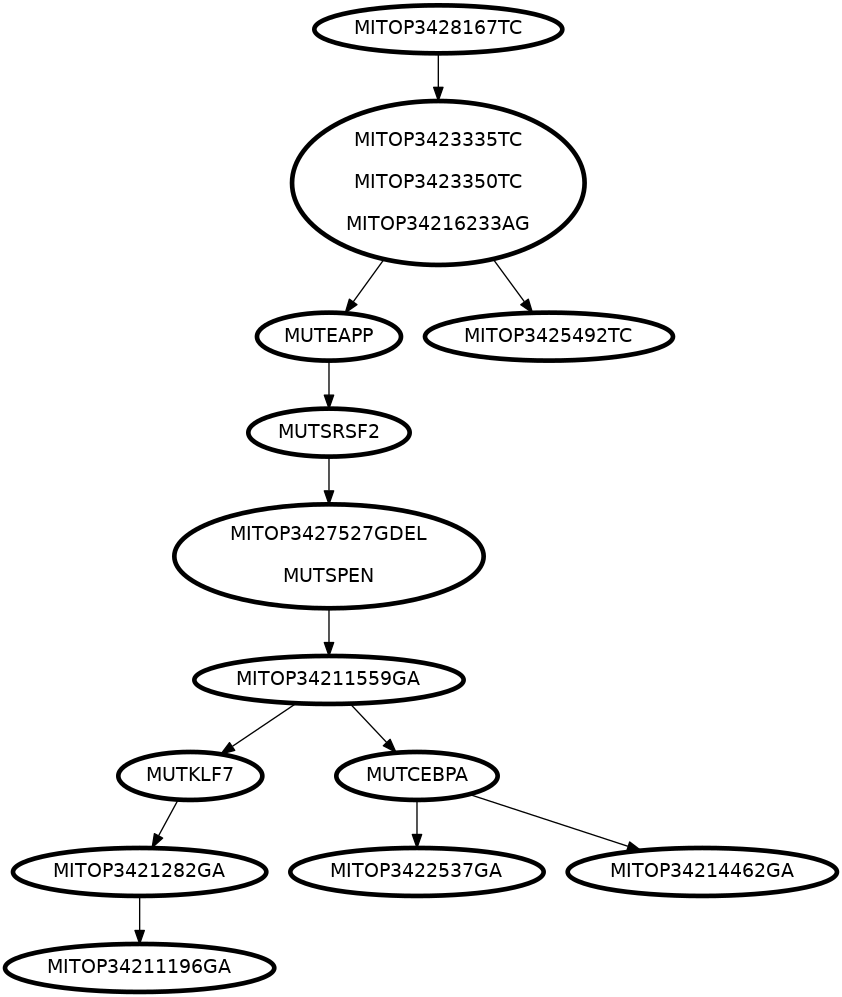

Supplement: Supplementary file 8 — Supplementary Data 5 [file 41467_2021_21650_MOESM8_ESM.zip › P342_002.png]

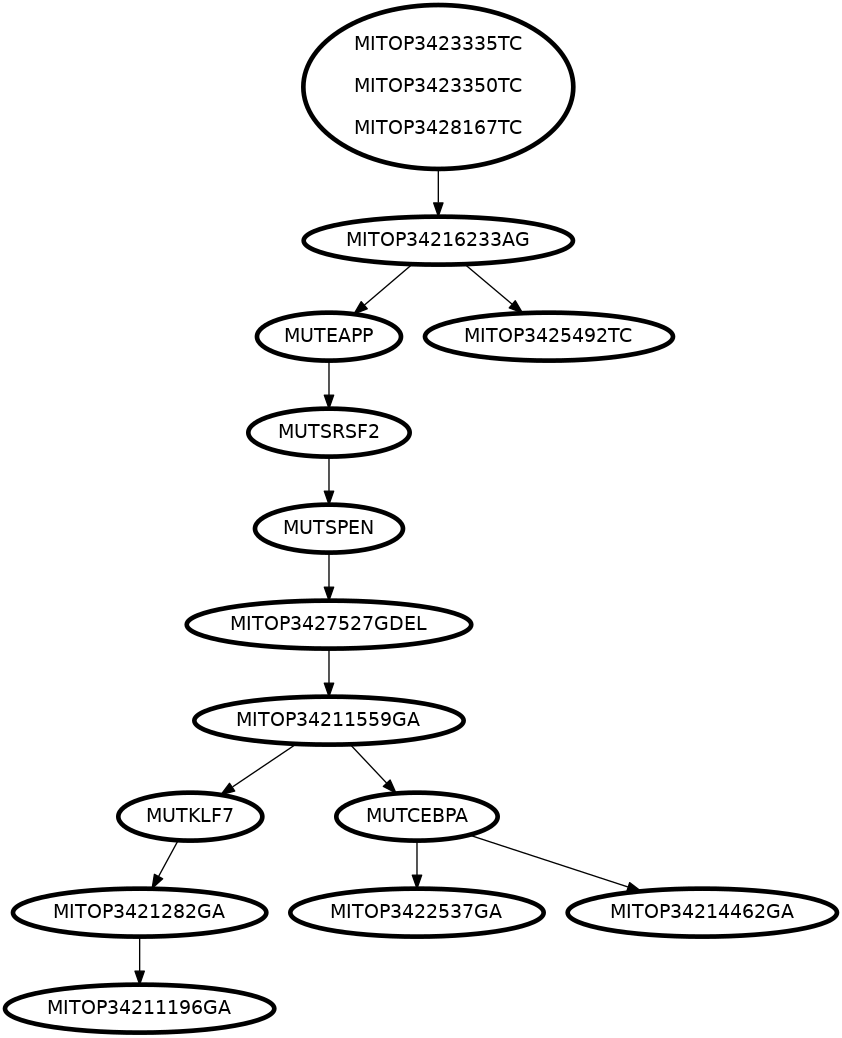

Supplement: Supplementary file 8 — Supplementary Data 5 [file 41467_2021_21650_MOESM8_ESM.zip › P342_003.png]

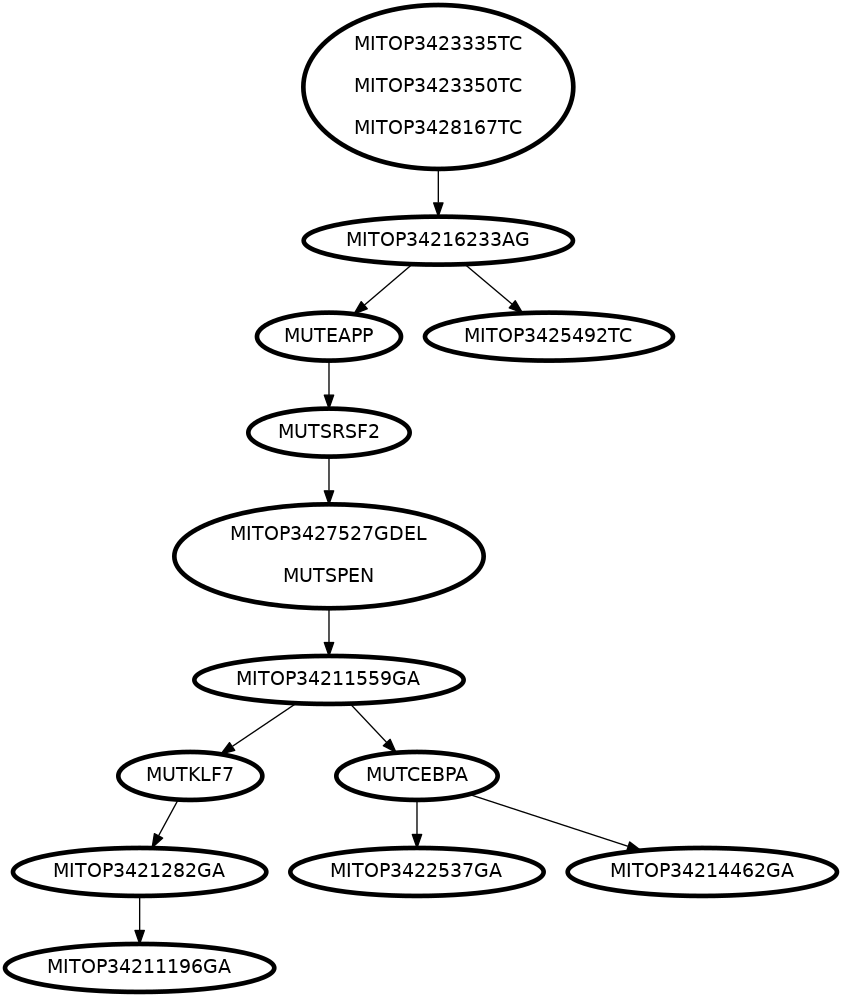

Supplement: Supplementary file 8 — Supplementary Data 5 [file 41467_2021_21650_MOESM8_ESM.zip › P342_004.png]

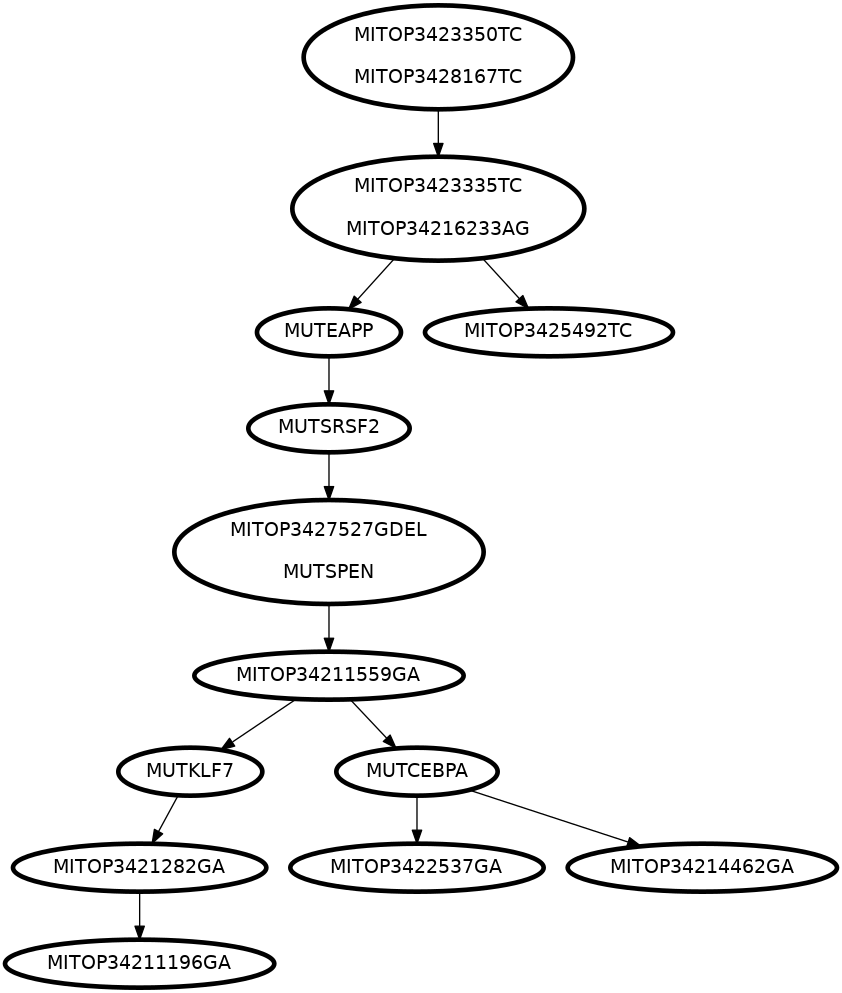

Supplement: Supplementary file 8 — Supplementary Data 5 [file 41467_2021_21650_MOESM8_ESM.zip › P342_010.png]

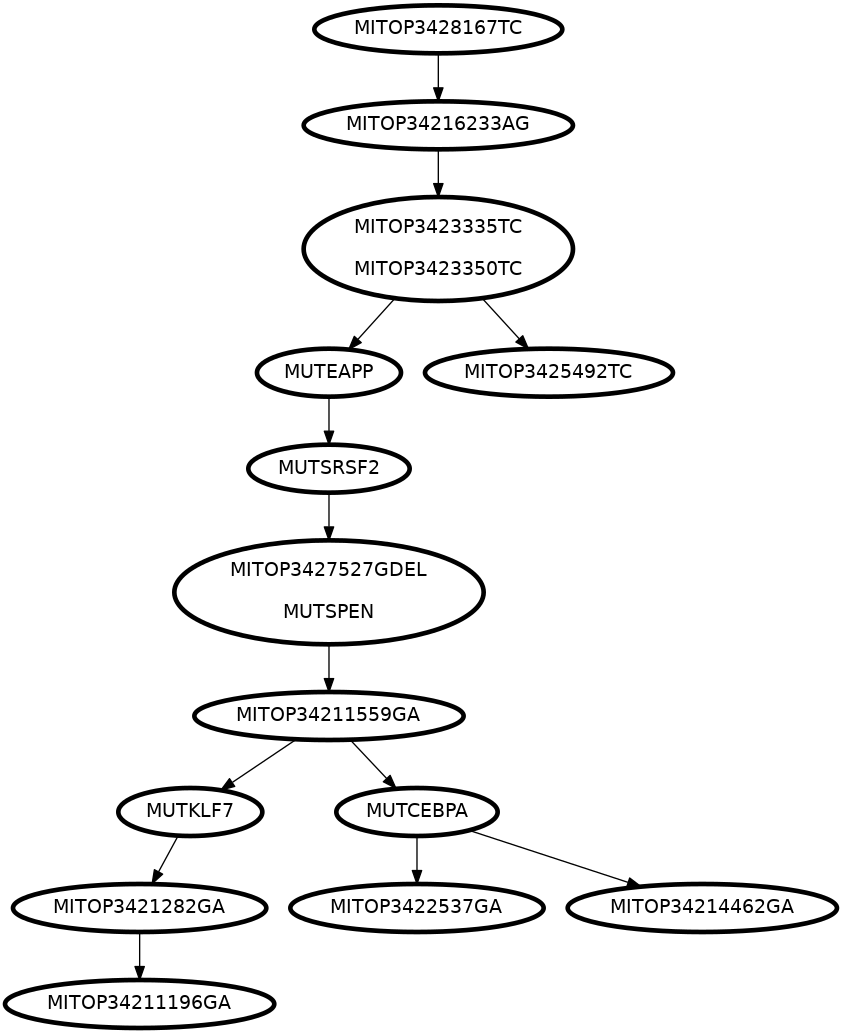

Supplement: Supplementary file 8 — Supplementary Data 5 [file 41467_2021_21650_MOESM8_ESM.zip › P342_012.png]

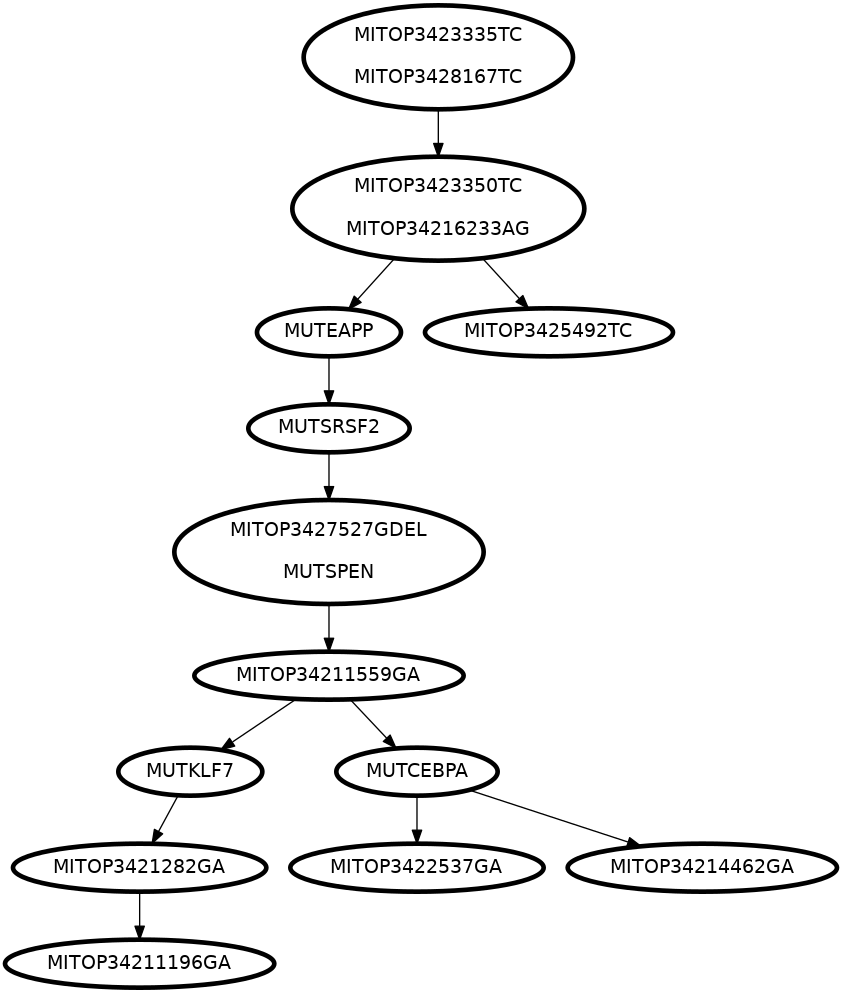

Supplement: Supplementary file 8 — Supplementary Data 5 [file 41467_2021_21650_MOESM8_ESM.zip › P342_013.png]

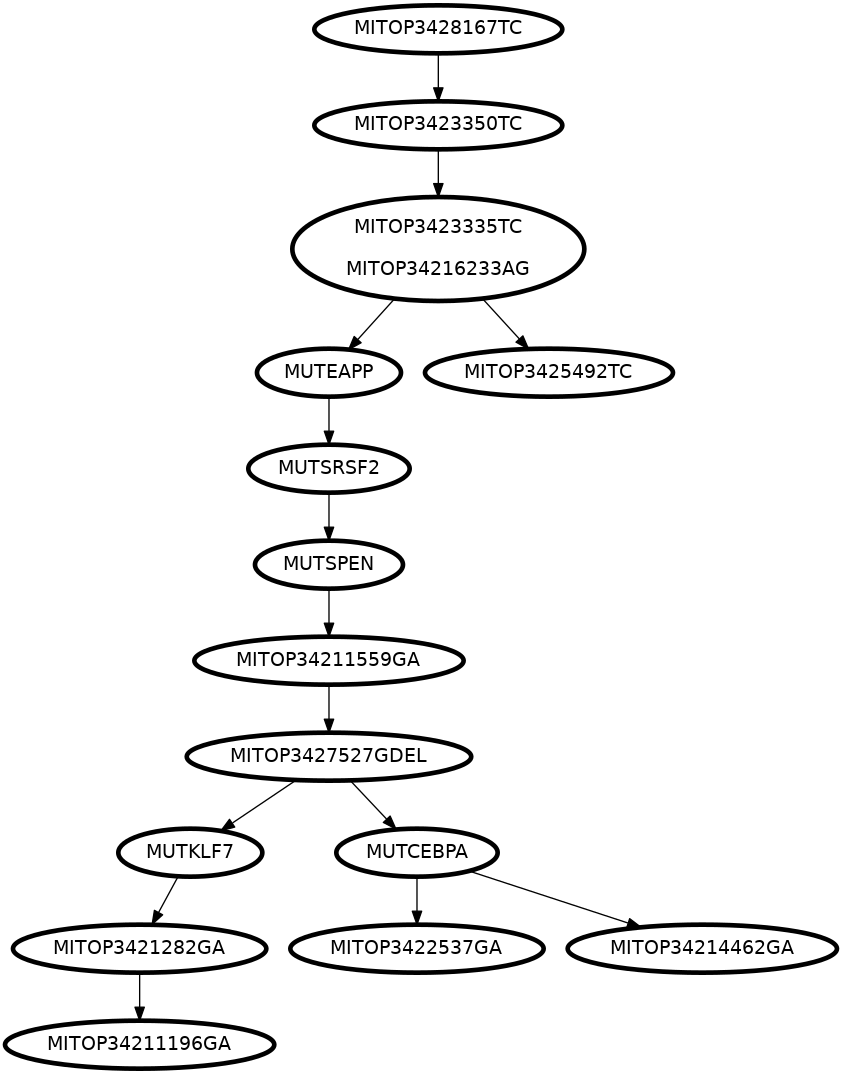

Supplement: Supplementary file 8 — Supplementary Data 5 [file 41467_2021_21650_MOESM8_ESM.zip › P342_016.png]

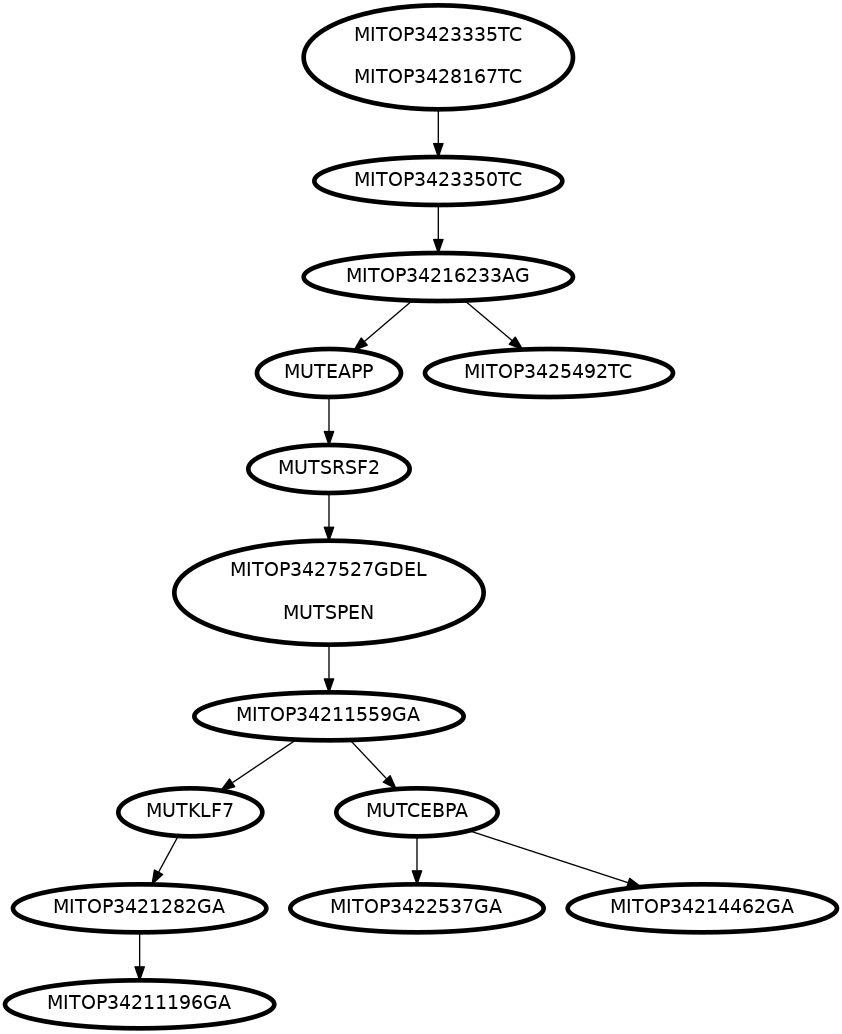

Supplement: Supplementary file 8 — Supplementary Data 5 [file 41467_2021_21650_MOESM8_ESM.zip › P342_024.png]

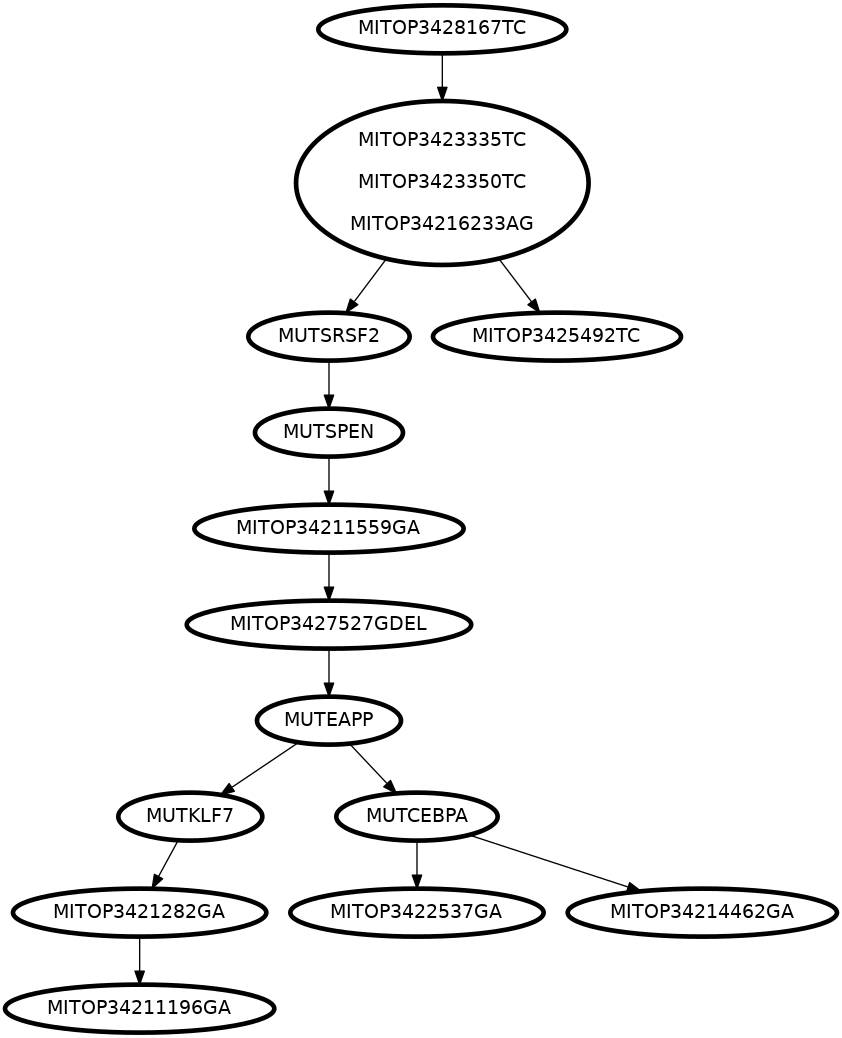

Supplement: Supplementary file 8 — Supplementary Data 5 [file 41467_2021_21650_MOESM8_ESM.zip › P342_027.png]

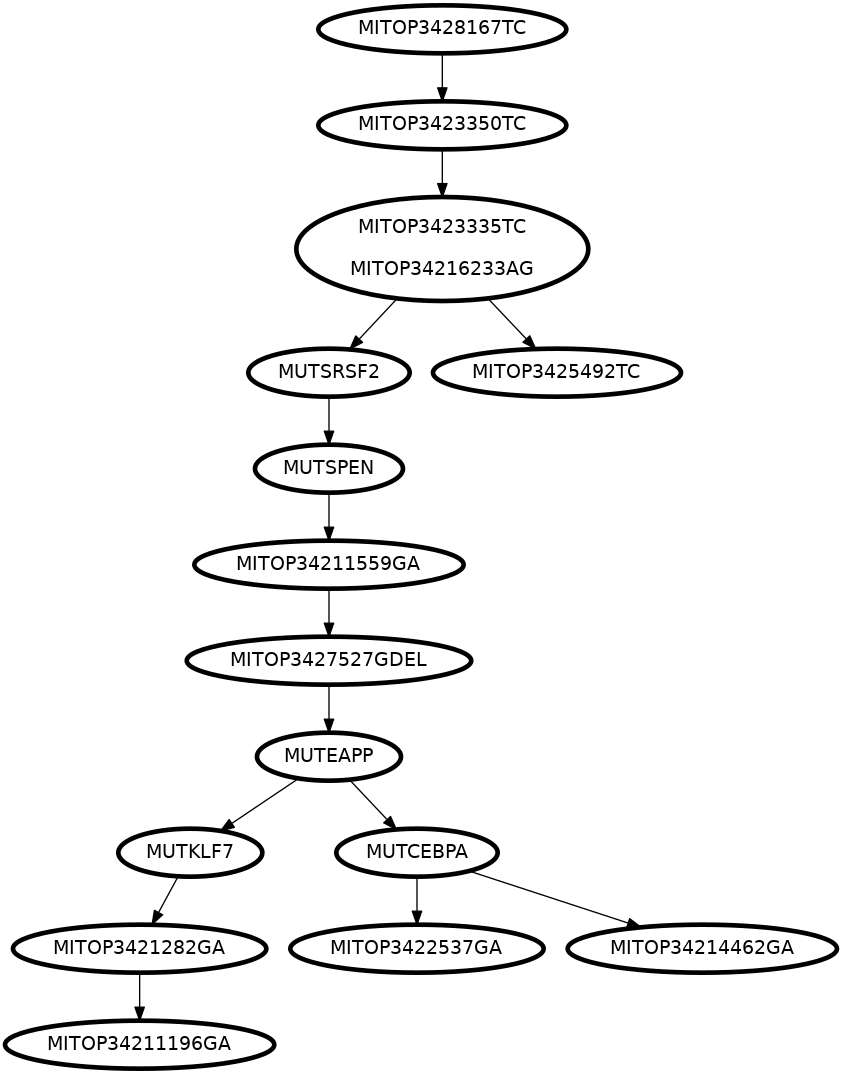

Supplement: Supplementary file 8 — Supplementary Data 5 [file 41467_2021_21650_MOESM8_ESM.zip › P342_034.png]

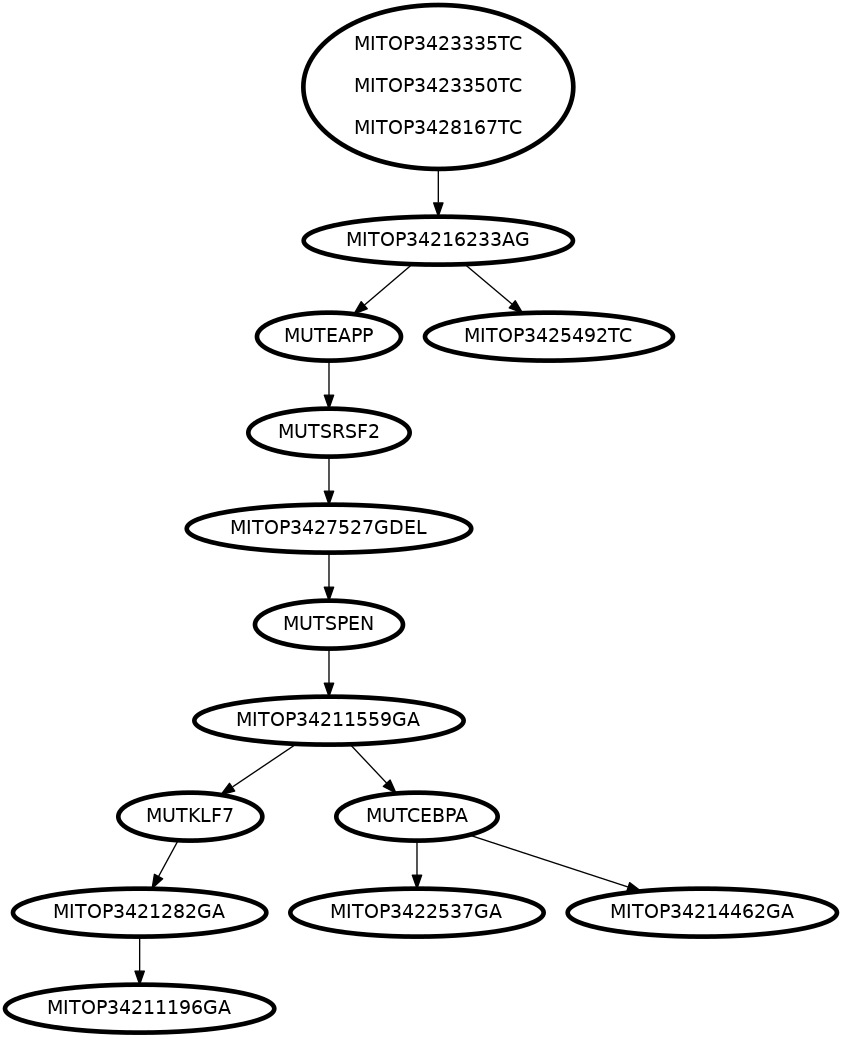

Supplement: Supplementary file 8 — Supplementary Data 5 [file 41467_2021_21650_MOESM8_ESM.zip › P342_035.png]

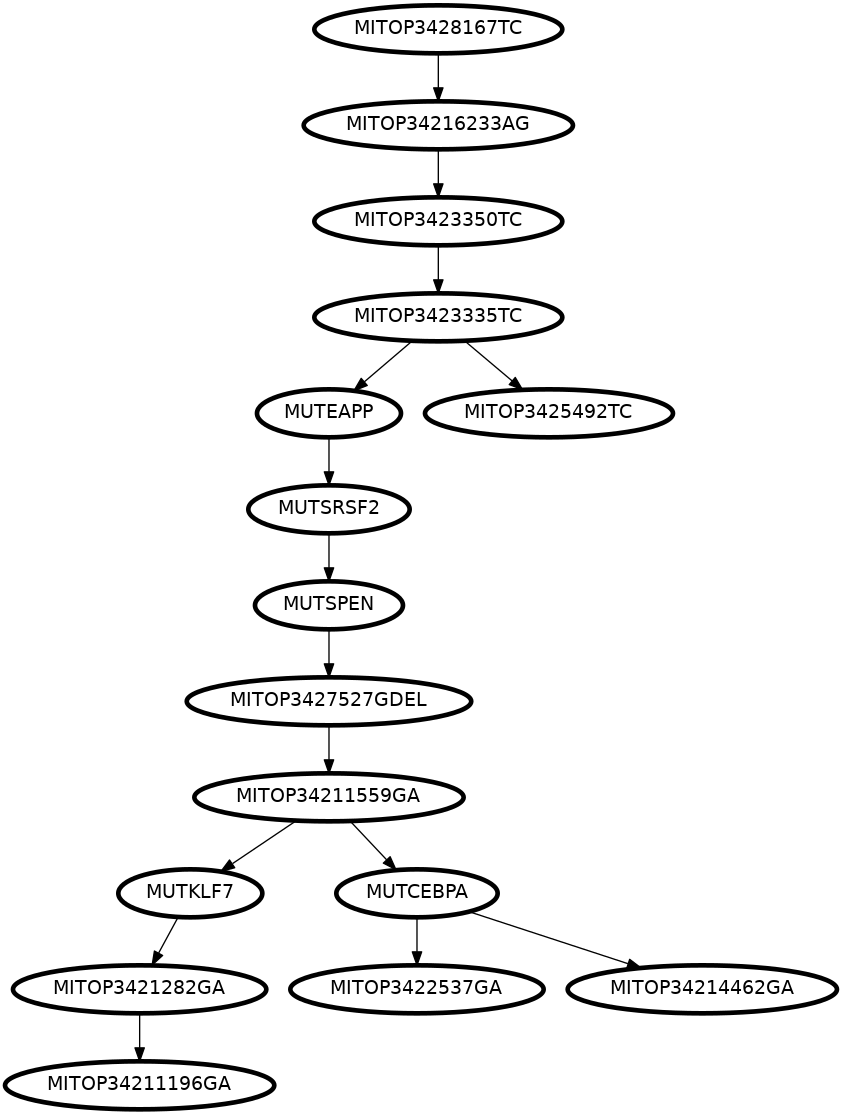

Supplement: Supplementary file 8 — Supplementary Data 5 [file 41467_2021_21650_MOESM8_ESM.zip › P342_037.png]

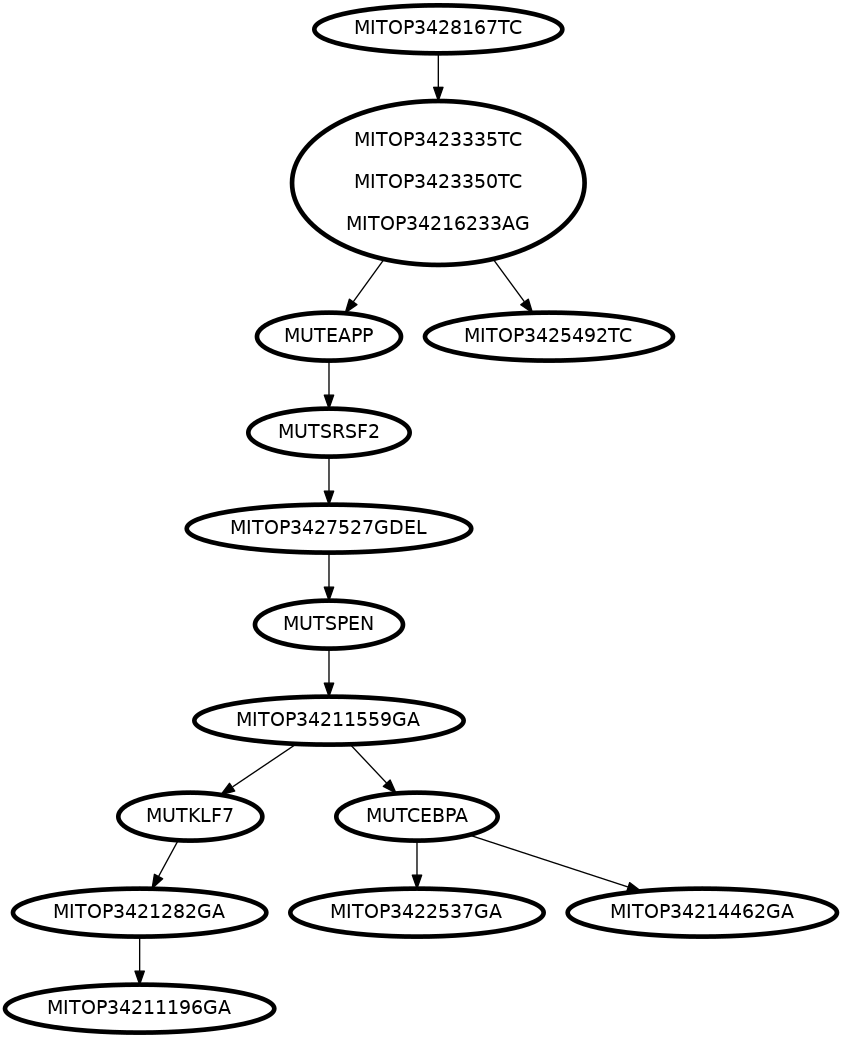

Supplement: Supplementary file 8 — Supplementary Data 5 [file 41467_2021_21650_MOESM8_ESM.zip › P342_039.png]

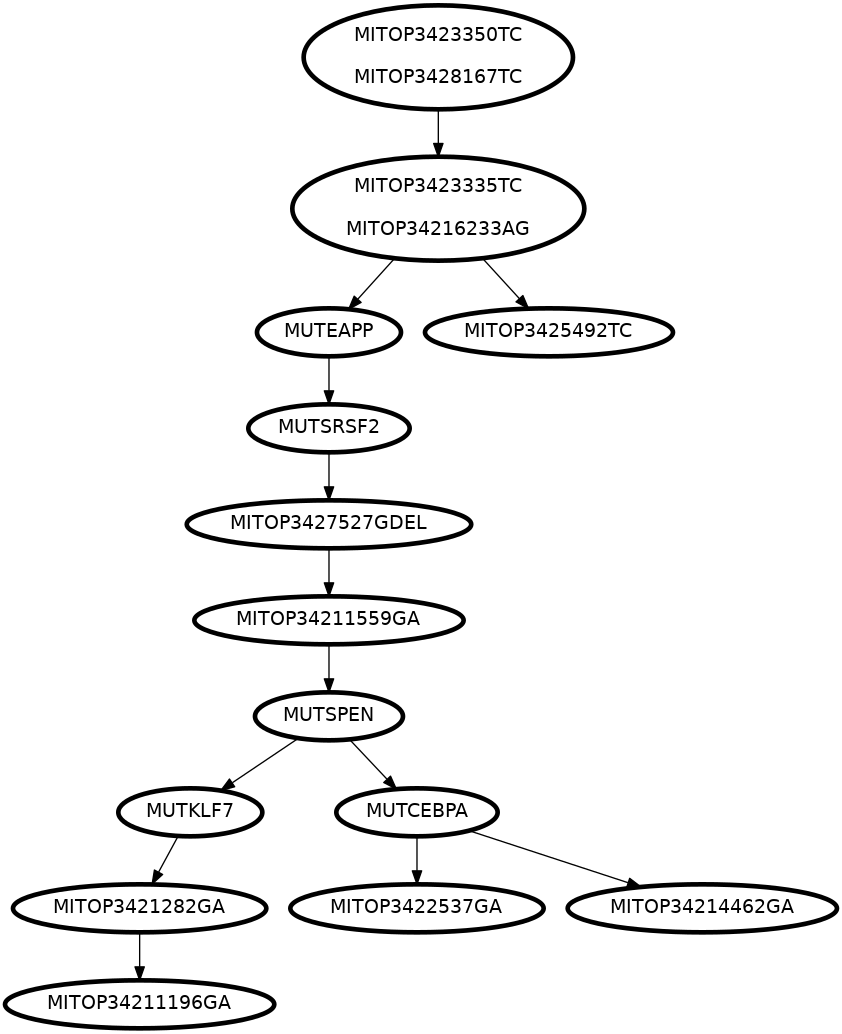

Supplement: Supplementary file 8 — Supplementary Data 5 [file 41467_2021_21650_MOESM8_ESM.zip › P342_040.png]

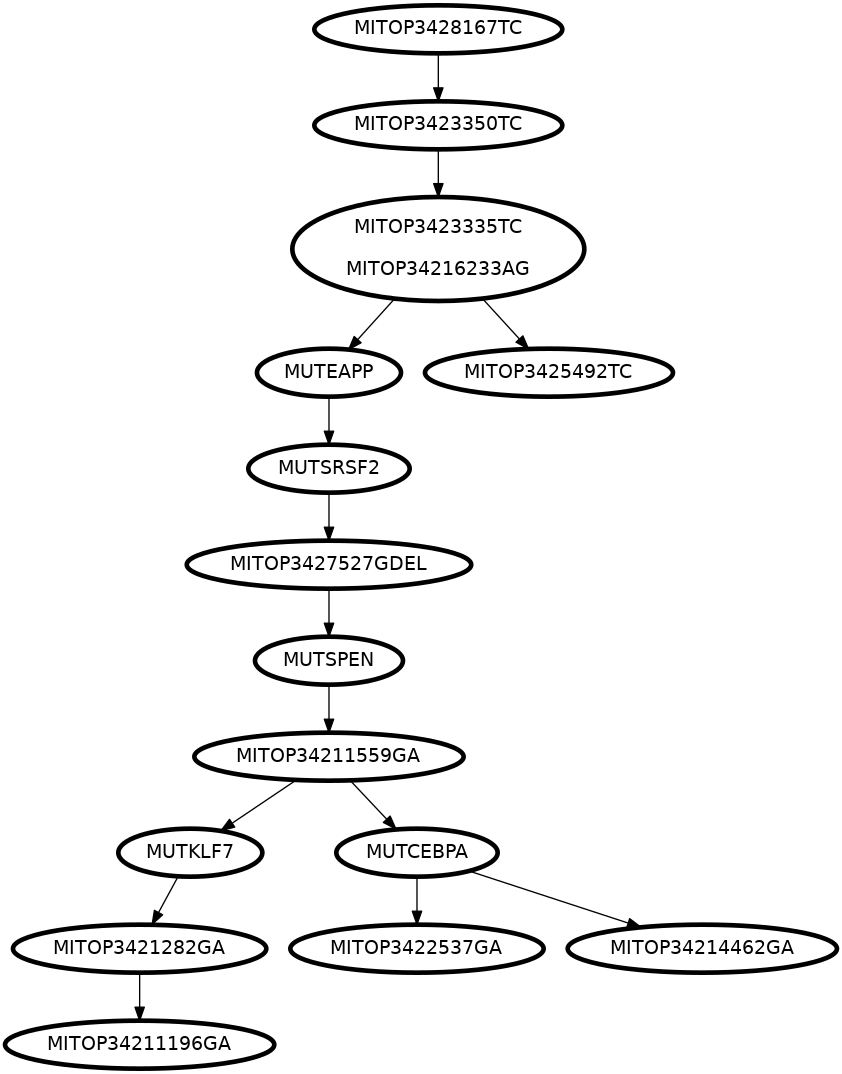

Supplement: Supplementary file 8 — Supplementary Data 5 [file 41467_2021_21650_MOESM8_ESM.zip › P342_059.png]

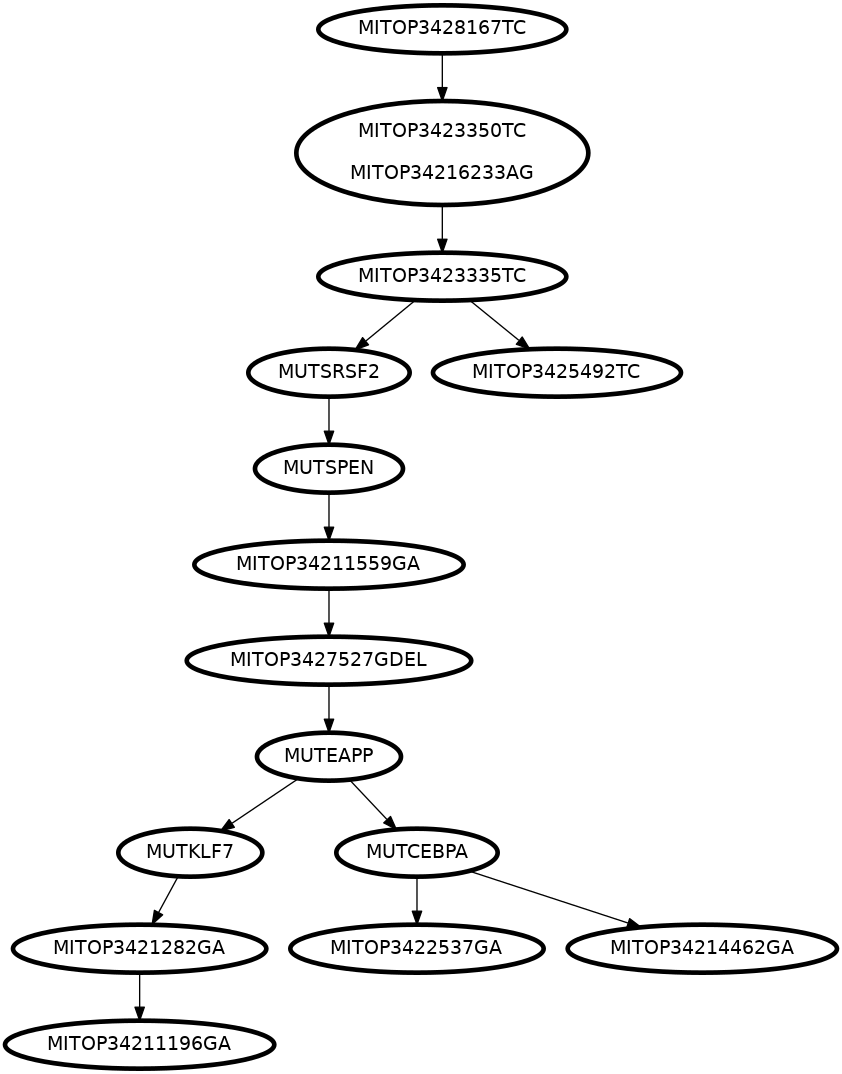

Supplement: Supplementary file 8 — Supplementary Data 5 [file 41467_2021_21650_MOESM8_ESM.zip › P342_061.png]

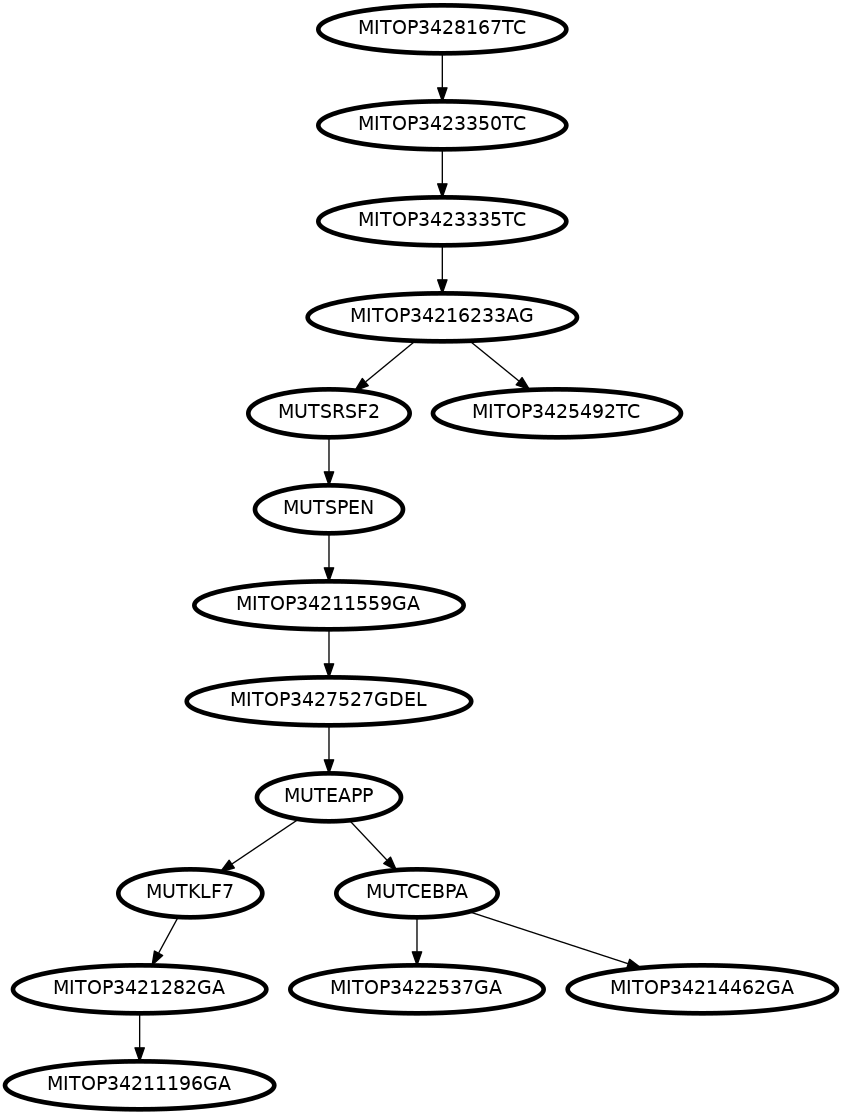

Supplement: Supplementary file 8 — Supplementary Data 5 [file 41467_2021_21650_MOESM8_ESM.zip › P342_062.png]

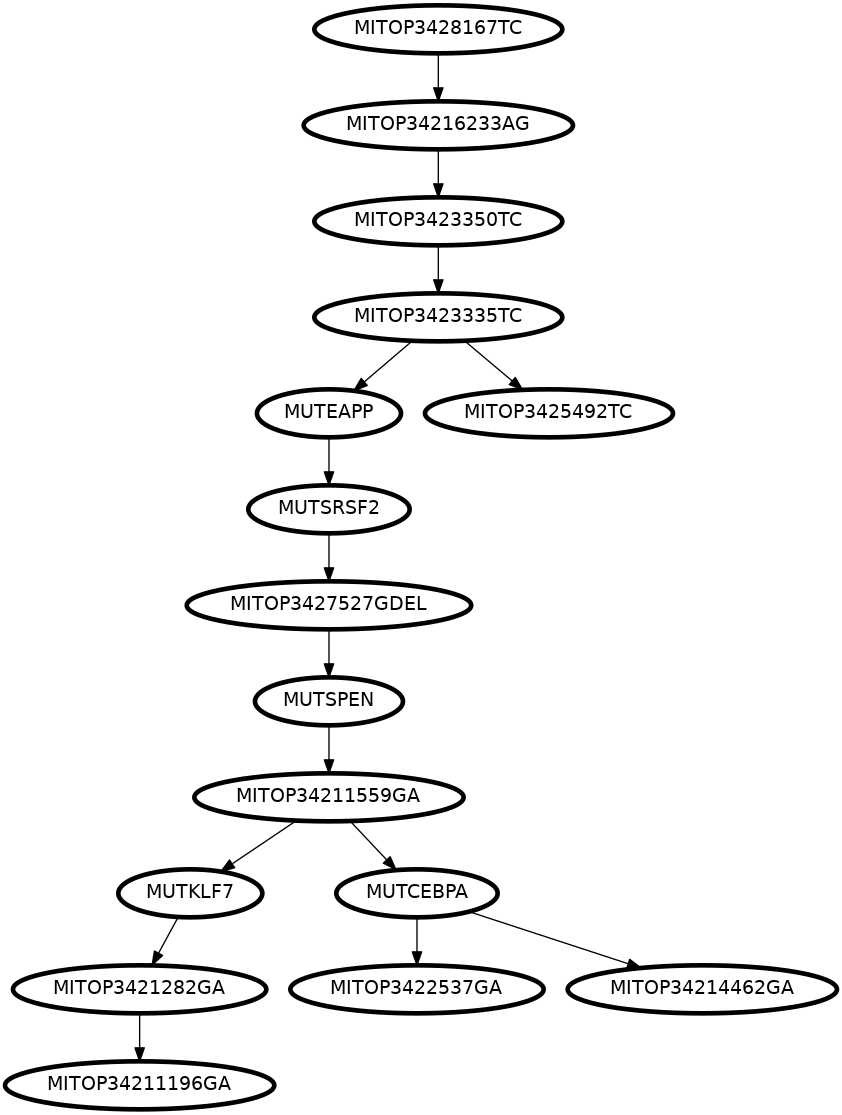

Supplement: Supplementary file 8 — Supplementary Data 5 [file 41467_2021_21650_MOESM8_ESM.zip › P342_065.png]

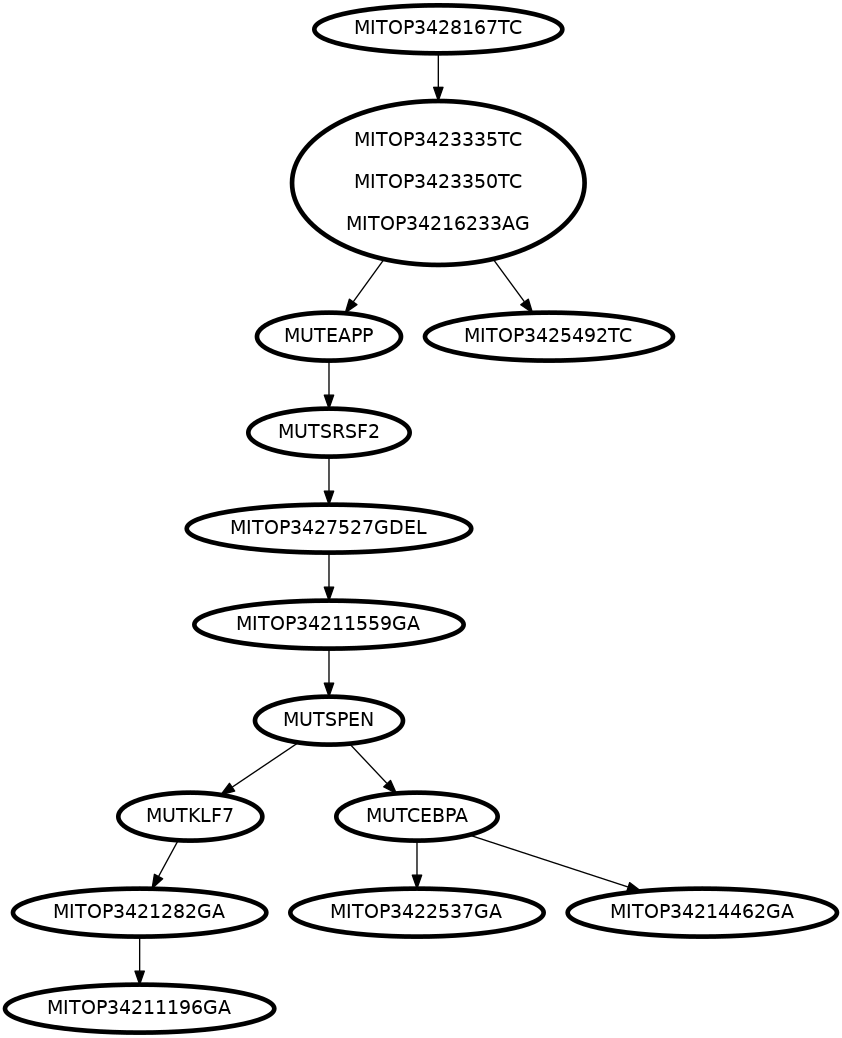

Supplement: Supplementary file 8 — Supplementary Data 5 [file 41467_2021_21650_MOESM8_ESM.zip › P342_066.png]

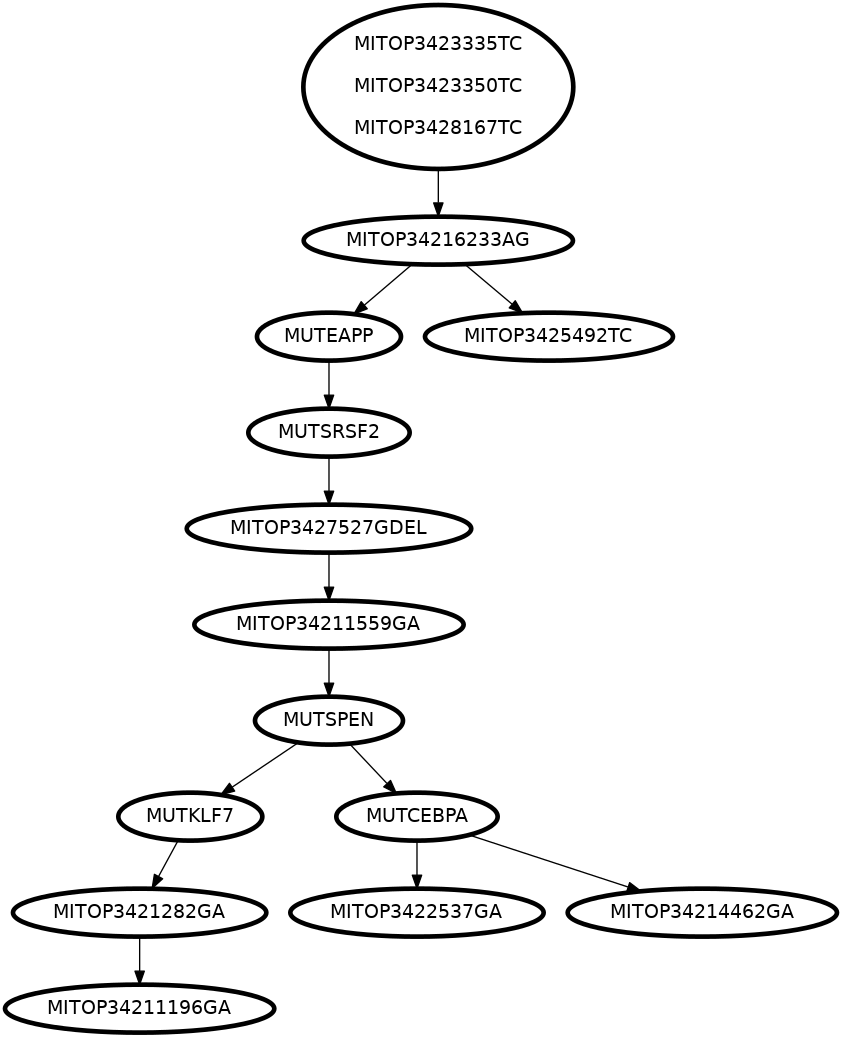

Supplement: Supplementary file 8 — Supplementary Data 5 [file 41467_2021_21650_MOESM8_ESM.zip › P342_067.png]

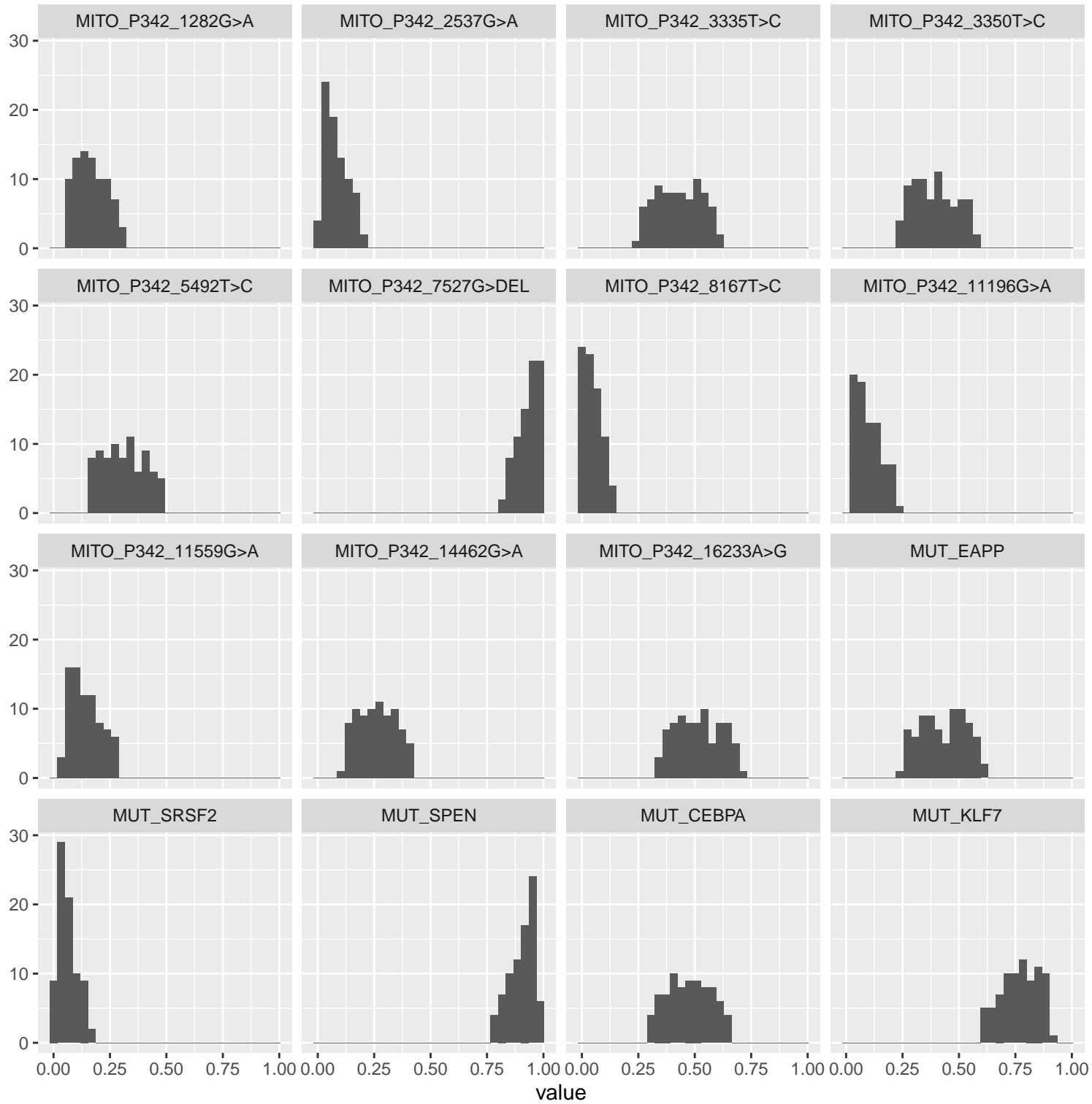

Supplement: Supplementary file 8 — Supplementary Data 5 [file 41467_2021_21650_MOESM8_ESM.zip › 007_param_ranges_lhs.pdf]
