# Supplementary material for: Identification of leukemic and pre-leukemic stem cells by clonal tracking from single-cell transcriptomics
Source: Nat Commun. 2021 Mar 1;12:1366. doi: 10.1038/s41467-021-21650-1 (PMC7921413; doi:10.1038/s41467-021-21650-1)
Supplement: Supplementary file 9 — Reporting summary [file 41467_2021_21650_MOESM9_ESM.pdf]

nature research

Corresponding author(s): Lars Velten, Lars Steinmetz
Last updated by author(s): Jan 20, 2021

Reporting Summary

Nature Research wishes to improve the reproducibility of the work that we publish. This form provides structure for consistency and transparency in reporting. For further information on Nature Research policies, see our Editorial Policies and the Editorial Policy Checklist.

Statistics

For all statistical analyses, confirm that the following items are present in the figure legend, table legend, main text, or Methods section.

n/a	Confirmed	
☐	☒	The exact sample size (*n*) for each experimental group/condition, given as a discrete number and unit of measurement
☐	☒	A statement on whether measurements were taken from distinct samples or whether the same sample was measured repeatedly
☐	☒	The statistical test(s) used AND whether they are one- or two-sided<br>*Only common tests should be described solely by name; describe more complex techniques in the Methods section.*
☐	☒	A description of all covariates tested
☐	☒	A description of any assumptions or corrections, such as tests of normality and adjustment for multiple comparisons
☐	☒	A full description of the statistical parameters including central tendency (e.g. means) or other basic estimates (e.g. regression coefficient) AND variation (e.g. standard deviation) or associated estimates of uncertainty (e.g. confidence intervals)
☐	☒	For null hypothesis testing, the test statistic (e.g. *F*, *t*, *r*) with confidence intervals, effect sizes, degrees of freedom and *P* value noted<br>*Give P values as exact values whenever suitable.*
☒	☐	For Bayesian analysis, information on the choice of priors and Markov chain Monte Carlo settings
☐	☒	For hierarchical and complex designs, identification of the appropriate level for tests and full reporting of outcomes
☒	☐	Estimates of effect sizes (e.g. Cohen's *d*, Pearson's *r*), indicating how they were calculated

Our web collection on statistics for biologists contains articles on many of the points above.

Software and code

Policy information about availability of computer code

Data collection	For primer design, a custom pipeline was used. The pipeline is made available on Git (https://git.embl.de/velten/PrimerDesign).
Data analysis	Analysis routines and reproduction instructions are available in the mitoClone package at https://github.com/veltenlab/mitoClone . 3rd party tools:<br>python 3.7.8 with packages scanorama (1.6) and PhISCS (1.0)<br>R version 3.6.2 with the following packages, in format Package Name_Version<br>[1] STEMNET_0.1 Seurat_3.1.4 scran_1.14.6 scmap_1.8.0<br>[5] scater_1.14.6 scales_1.1.0 reshape2_1.4.3 plyr_1.8.6<br>[9] pheatmap_1.0.12 MAST_1.12.0 gridExtra_2.3 glmnet_3.0-2<br>[13] Matrix_1.2-18 ggrepel_0.8.1 ggplot2_3.2.1 flowCore_1.52.1<br>[17] biomaRt_2.45.7 batchelor_1.2.4 SingleCellExperiment_1.8.0 SummarizedExperiment_1.16.1<br>[21] DelayedArray_0.12.2 BiocParallel_1.20.1 matrixStats_0.55.0 Biobase_2.46.0<br>[25] GenomicRanges_1.38.0 GenomeInfoDb_1.22.0 IRanges_2.20.2 S4Vectors_0.24.3<br>[29] BiocGenerics_0.32.0

For manuscripts utilizing custom algorithms or software that are central to the research but not yet described in published literature, software must be made available to editors and reviewers. We strongly encourage code deposition in a community repository (e.g. GitHub). See the Nature Research guidelines for submitting code & software for further information.

Data

Policy information about availability of data

All manuscripts must include a data availability statement. This statement should provide the following information, where applicable:

- Accession codes, unique identifiers, or web links for publicly available datasets
- A list of figures that have associated raw data
- A description of any restrictions on data availability

Count tables and other processed data necessary to reproduce all analysis from the manuscript are deposited in figshare with DOI 10.6084/m9.figshare.12382685.v1. Raw sequencing data are deposited under a Data Access Agreement to protect patient privacy in the European Genome-Phenome Archive with the accession id EGAS00001003414. Sequencing data from the healthy individuals are deposited in GEO with the accession id GSE75478. The source data underlying Figures 1f, 5d, 5h and Supplementary Figures 8c-e are provided as a Source Data file. All the other data supporting the findings of this study are available within the article and its supplementary information files and from the corresponding author upon reasonable request.

Field-specific reporting

Please select the one below that is the best fit for your research. If you are not sure, read the appropriate sections before making your selection.

- [x] Life sciences
- [ ] Behavioural & social sciences
- [ ] Ecological, evolutionary & environmental sciences

For a reference copy of the document with all sections, see nature.com/documents/nr-reporting-summary-flat.pdf

Life sciences study design

All studies must disclose on these points even when the disclosure is negative.

Sample size	n = 5228 cells were included in the study. Number of cells covered per patient (618 - 1430) follows best practices in well-based single-cell RNA-seq studies of the hematopoietic system (Velten et al., Nature Cell Biology 19:271-281, Nestorowa et al., Blood 128:e20-31) and can further be justified as follows:<br>Power for cell type discovery: Cell types were covered with 37 to 667 cells. A minimum of 10-50 cells are required to identify cell types, depending on the degree of similarity to other cell types. Cell types present at below 0.2%-1% of all cells sampled may therefore be missed.<br>Power for clone discovery: Clones marked by at least one mitochondrial variant were covered with at least 25 cells, corresponding to 1.7% of cells in that patient. Clones present at below 1-5% of cells may therefore be missed.
Data exclusions	In the MutaSeq experiments with Patients 1 to Patients 4, cells with less than 500 distinct genes observed and genes that appeared in less than 5 cells were removed. This criterion was pre-established and is customary in the field, see Luecken & Theis, Mol Syst Biol 15: e8746. The rationale for this data exclusion is that in single cell RNA-seq expeirments, some cells are of poor sample quality (e.g. apoptotic or damaged cells) and can therefore not be used to retrieve meaningful information.
Replication	Experiment was replicated across 4 patients, and profound patient-to-patient heterogeneity was observed. All attempts at replication were successful.
Randomization	Not applicable: There were no control or treatment groups.
Blinding	Not applicable: There were no control or treatment groups.

Reporting for specific materials, systems and methods

We require information from authors about some types of materials, experimental systems and methods used in many studies. Here, indicate whether each material, system or method listed is relevant to your study. If you are not sure if a list item applies to your research, read the appropriate section before selecting a response.

Materials & experimental systems

n/a	Involved in the study	
[ ]	[x]	Antibodies
[x]	[ ]	Eukaryotic cell lines
[x]	[ ]	Palaeontology and archaeology
[x]	[ ]	Animals and other organisms
[ ]	[x]	Human research participants
[x]	[ ]	Clinical data
[x]	[ ]	Dual use research of concern

Methods

n/a	Involved in the study	
[x]	[ ]	ChIP-seq
[ ]	[x]	Flow cytometry
[x]	[ ]	MRI-based neuroimaging

Antibodies

Antibodies used

See Supplementary Table 1.
Antigen,Used for,Clone,Fluorochrome,Company,Dilution,Catalogue Number
CD135,Index Sort,4G8,PE,BD Pharmingen,1:20,558996
CD15,Colony Classification,W6D3,Alexa700,BioLegend,1:100,323026
CD19,Colony Classification,HIB19,eFluor 450,eBioscience,1:80,48-0199-42
CD19,Index Sort,HIB19,APC,eBioscience,1:20,17-0199-42
CD20,Index Sort,2H7,APC,BD Pharmingen,1:20,559776
CD235a,Colony Classification/Index Sort,HIR2,APC,BD Pharmingen,1:30,551336
CD33,Colony Classification,WM-53,PE-Cy7,eBioscience,1:200,12-0338-42,12-0338-42
CD33,Index Sort,WM53,BV421,BioLegend,1:100,303416
CD34,Colony Classification/Index Sort,4H11,APC-eFluor 780,eBioscience,1:30,47-0349-42
CD38,Index Sort,HIT2,Alexa 700,eBioscience,1:30,56-0389-42
CD4,Index Sort,RPA-T4,APC,BD Pharmingen,1:20,555349
CD41a,Colony Classification,HIP8,FITC,eBioscience,1:200,11-0419-42
CD41a,Index Sort,HIP8,APC,eBioscience,1:30,17-0419-42
CD45,Colony Classification/Mesenchymal sort,HI30,PE,eBioscience,1:200,12-0459-42
CD45RA,Index Sort,HI100,FITC,BioLegend,1:20,983002
CD66b,Colony Classification,G10F5,PerCP/Cy5.5,BioLegend,1:100,305108
CD8,Index Sort,RPA-T8,APC,BD Pharmingen,1:20,555369
CD90,Index Sort,5E10,PE-Cy5,BD Pharmingen,1:20,555597
GPR56,Index Sort,CG4,PE-Cy7,BioLegend,1:20,358206
Tim3,Index Sort,F38-2E2,BV605,BioLegend,1:50,345018
CD105,Mesenchymal sort,43A3,FITC,BioLegend,1:30,323204

Validation

Antibodies were validated by the manufacturer who provided references on their websites using the catalog number provided above:
Biolegend - https://www.biolegend.com/
BD - https://www.bdbiosciences.com/
eBioscience: https://www.thermofisher.com/

Human research participants

Policy information about studies involving human research participants

Population characteristics

Male AML patients aged 18-78 years. Ethnicity unknown.

Recruitment

Participants were recruited following standard procedures in the University Hospital Mannheim and Heidelberg University Hospital: AML patients at first diagnosis were asked if they would voluntarily make parts of the material taken for diagnostic purposes available for research (informed written consent). Samples were further selected based on the availability of genomic DNA coming from both AML and healthy tissue from the patients. There is no reason to believe that these selection criteria would introduce a selection bias or otherwise impact the results.

Ethics oversight

Ethics commission of the medical faculties Heidelberg and Heidelberg-Mannheim of the University of Heidelberg; Bioethics Internal Advisory Committee (BIAC) at EMBL and the CRG bioethics committee (CEIC-Parc de Salut Mar)

Note that full information on the approval of the study protocol must also be provided in the manuscript.

Flow Cytometry

Plots

Confirm that:

- [x] The axis labels state the marker and fluorochrome used (e.g. CD4-FITC).
- [x] The axis scales are clearly visible. Include numbers along axes only for bottom left plot of group (a 'group' is an analysis of identical markers).
- [x] All plots are contour plots with outliers or pseudocolor plots.
- [x] A numerical value for number of cells or percentage (with statistics) is provided.

Methodology

Sample preparation

Bone marrow mononuclear cells were stained for 30 minutes on ice according to standard protocols. For single-cell liquid cultures and MutaSeq, cells were stained with fluorescent-labelled antibodies against lineage markers (CD4, CD8, CD19, CD20, CD41a, CD235a) and additional markers (CD45RA, CD135, GPR56, CD34, CD38, CD90, CD33, Tim3), and sorted according to the gating scheme illustrated in Figure S2.

Instrument

BD FACS Fusion (BD Biosciences) equipped with 405nm, 488nm, 561nm and 640nm lasers were used.

Software

FlowJo v10 TreeStar, R 3.6.2 (flowCore 1.52.1)

Cell population abundance

Composition of sorted populations in terms of cell types was determined post sort using single cell transcirptomics, see also Fig. 3 and Supplementary Fig. 2. The below table specifies the composition of each gate (columns) in terms of cell types (rows).

CD34-Lin-,CD34-Lin+,CD34+Lin-,Lin-,TBM
Erythroid precursors,0,0.035,0.041,0.007,0.007
MEP,0,0.002,0.037,0.013,0.004
Heterogeneous progenitors,0.002,0,0.182,0.207,0.061
CD34- Blasts (Calprotectin-AZU1+),0.169,0.113,0.011,0.017,0.169
Neutrophil precursors,0.002,0.002,0.026,0,0.002
Mitotic HSPCs (G2/M),0,0,0.031,0.01,0.002
Lymphoid precursors,0.006,0.011,0.08,0.003,0.018
Healthy HSPCs,0,0.003,0.121,0.013,0.008
NK and T cells,0.006,0.011,0.006,0,0.015
CD34+ Blasts,0,0,0.283,0.254,0.058
CD34- Blasts (Calprotectin+AZU1+),0.168,0.164,0.019,0.003,0.186
CD34- Blasts (HBZ+),0.142,0.161,0.103,0.003,0.062
CD34- Blasts (Intermediate),0.063,0.172,0.012,0.023,0.076
CD34- Blasts (Calprotectin+AZU1-),0.343,0.211,0.013,0.057,0.154
CD34- Blasts (Unclear),0.09,0.012,0.019,0,0.002
Other T/NK cells,0.002,0.011,0.005,0.04,0.008
Cytotoxic T-cells,0.002,0.025,0.003,0.097,0.049
Effector memory T-cells,0.002,0.02,0.002,0.054,0.029
Central memory T-cells,0,0.049,0.005,0.017,0.057
NK cells,0.006,0,0.001,0.181,0.032

Gating strategy

Cells were sorted as shown in Supplementary Fig. 2. In detail, first cells were separated from debris by FSC/SSC segregation. Afterwards, duplets were avoided by plotting FSC-A vs FSC-H and SSC-A vs FSC-H. Last, a combination of lineage markers (to distinguish mature and immature cells) was plotted against CD34 to further segregate immature from stem cells. Positivity and negativity to this markers was established due to characteristic bimodal staining of such proteins and adjusted in a sample-specific manner.

☒ Tick this box to confirm that a figure exemplifying the gating strategy is provided in the Supplementary Information.
